# Intracranial hemorrhage prediction in acute ischemic stroke patients with anterior circulation tandem lesions following endovascular thrombectomy

**DOI:** 10.3389/fneur.2025.1598203

**Published:** 2025-08-29

**Authors:** Wenqing Tian, Li Zhou, Yueqi Zhang

**Affiliations:** ^1^Department of Neurology, Weifang People’s Hospital, Shandong Second Medical University, Weifang, China; ^2^School of Clinical Medicine, Shandong Second Medical University, Weifang, China

**Keywords:** tandem lesions, thrombectomy, hemorrhage, prognosis, stroke

## Abstract

**Background:**

Acute ischemic stroke (AIS) patients with anterior circulation tandem lesions (TL) face a heightened risk of hemorrhage following endovascular thrombectomy (EVT). Predictive models specifically for this complication in the TL population are currently lacking.

**Methods:**

This retrospective cohort study analyzed 200 AIS patients with anterior circulation TL who underwent EVT. Least Absolute Shrinkage and Selection Operator regression was used for feature selection. Multivariable logistic regression (LR) models predicting intracranial hemorrhage (ICH) and symptomatic intracranial hemorrhage (sICH) risk were developed and visualized as nomograms. Model discrimination was evaluated using the area under the receiver operating characteristic curve (AUC).

**Results:**

After EVT, ICH occurred in 92 patients (46%) and sICH in 24 patients (12%). The LR model for ICH identified diabetes [odd ratio (OR) 2.454, 95% CI 1.137–5.297], drinking history (OR 2.230, 95% CI 1.160–4.288), and lower modified Thrombolysis in Cerebral Infarction (mTICI) score (OR 0.547, 95% CI 0.311–0.961) as significant independent predictors (AUC = 0.712). The LR model for sICH identified lower Glasgow Coma Scale (GCS) score (OR 0.820, 95% CI 0.695–0.968), lower mTICI score (OR 0.398, 95% CI 0.182–0.868), and lower Alberta Stroke Program Early CT Score (ASPECTS) (OR 0.795, 95% CI 0.641–0.984) as significant independent predictors (AUC = 0.830). Nomograms effectively quantified the contribution of predictors to outcome probabilities.

**Conclusion:**

In AIS patients with anterior circulation TL undergoing EVT, diabetes, drinking history, and lower mTICI score independently predict ICH risk, while lower GCS score, lower mTICI score, and lower ASPECTS independently predict sICH risk. The nomograms provide practical tools for individualized risk assessment, aiding clinical decision-making and perioperative management in this high-risk cohort.

## 1 Introduction

Worldwide, Acute ischemic stroke (AIS) ranks as a crucial contributor of mortality and disability, with an increasing burden due to aging populations and rising incidence among younger individuals (1, 2). Tandem lesions (TL) are a more severe form of cerebrovascular disease, characterized by significant stenosis or occlusion in extracranial vessels, coupled with distal intracranial vessel occlusion (3, 4). Endovascular thrombectomy (EVT) has been proven to be beneficial for AIS patients with TL (5, 6). However, compared to patients with isolated stenosis, AIS patients with TL have a more complex pathological mechanism, greater difficulty in achieving vessel recanalization, and a worse prognosis. They also face an increased risk of intracranial hemorrhage (ICH) after EVT, along with higher rates of disability and mortality (3, 7–10).

ICH is a common complication following EVT that has been established as an independent risk factor affecting patient prognosis, and symptomatic intracranial hemorrhage (sICH) represents a more severe form (6, 10–12). Identifying risk factors influencing the occurrence of ICH and sICH after EVT in TL patients can aid clinicians in timely recognition and intervention, thereby controlling the progression of surgical outcomes. This can prevent situations where hemorrhage after EVT impacts antiplatelet therapy or necessitates surgical intervention due to sICH, both of which severely compromise patient outcomes (5, 9–11, 13, 14).

Currently, no predictive model exists for assessing the risk of ICH and sICH after EVT in TL patients. This study aims to construct a machine learning (ML) model to predict the risk of ICH and sICH following EVT in TL patients. We ultimately utilized a nomogram to intuitively display the relative importance of various independent variables in the model, facilitating the control of risk factors and improving patient prognosis.

## 2 Methods

### 2.1 Study cohort

We retrospectively collected data from AIS patients with TL who were admitted to Weifang People’s Hospital from various regions across the country between June 2020 and November 2024. This study aligns with the guiding principles of the Declaration of Helsinki. As a retrospective study with all data anonymized, it was exempt from the requirement for patient informed consent based on relevant ethical regulations. Informed consent was obtained from all patients prior to undergoing EVT.

The inclusion criteria were as follows: (1) Diagnosis of AIS by neurologists based on symptoms and imaging examinations; (2) Confirmation of hemodynamically significant obstruction (occlusion or stenosis ≥50%) in the extracranial segment of the anterior circulation large vessel, along with distal intracranial segment occlusion, via magnetic resonance angiography, computed tomography angiography, or digital subtraction angiography (3, 4); (3) Patients who underwent EVT. The exclusion criteria were as follows: (1) Preoperative existence of ICH; (2) History of coagulation disorders, platelet abnormalities, or thrombocytopenia; (3) Allergy to contrast agents; (4) Severe cardiac, hepatic, renal, or other organ dysfunction; (5) Patients deemed by researchers to interfere with the data interpretation of this study.

### 2.2 Data collection

All data were sourced from objective electronic medical records. Patients diagnosed with AIS who presented within the EVT treatment window (24 h) were immediately enrolled in our hospital’s Stroke Green Channel (15, 16). Preoperative non-contrast head computed tomography (CT) was used to assess the Alberta Stroke Program Early CT Score (ASPECTS), quantifying the infarct core volume before treatment (16–18). The decision to perform EVT was made at the discretion of experienced, standardized-trained interventional neurologists, with specific treatment modalities (including stent retriever thrombectomy, balloon angioplasty, or intraarterial thrombolysis) chosen based on the patient’s condition. All patients underwent head CT immediately after the procedure and within 24 h postoperatively. Persistent hyperdensity on non-contrast CT was used to distinguish ICH from contrast extravasation. Dual energy CT was also employed to identify hemorrhage or contrast extravasation (7, 12, 14, 19). Non-contrast CT was performed using a SIEMENS CT WKL scanner (Siemens Healthcare) with the following parameters: tube voltage 120 kV, tube current 273 mAs, slice thickness 0.6 mm. Dual energy CT was performed using a SIECT DRIVE YX scanner (Siemens Healthcare) with the following parameters: simultaneous imaging at 80 kV/248 mAs and 140 kV/124 mAs, slice thickness 0.6 mm. Raw spiral projection data were reconstructed into three sets: two corresponding to 80 and 140 kV, respectively, and a third representing a mixed-energy image simulating conventional 120 kV. Virtual non-contrast image and iodine overlay map were utilized to differentiate hemorrhage from contrast (14, 20). Two physicians independently reviewed the imaging findings. In cases of disagreement, a third physician determined the final result.

Collected baseline demographic data included body mass index (BMI), age, and sex. Preoperative clinical data included grade of hypertension, diabetes, smoking history, drinking history, prior anticoagulant use, prior antiplatelet use, intravenous thrombolysis (bridging), time from symptom onset to groin puncture (onset to puncture time), National Institutes of Health Stroke Scale (NIHSS) score, Glasgow Coma Scale (GCS) score, admission systolic blood pressure, and ASPECTS. Procedural details included number of stent retriever device passes (retriever pass count), number of balloon angioplasty to dilate intracranial vessel (angioplasty count), intraarterial thrombolysis, and modified Thrombolysis in Cerebral Infarction (mTICI) score. Among the variable “grade of hypertension,” eight missing values were imputed using the mode (grade 3) among the population with hypertension, as these patients were only described as having hypertension without a specified grade. Missing BMI values (*n* = 6) were imputed using sex-specific averages (males: 24.681; females: 24.097).

Clinical outcomes were defined as any ICH and sICH after EVT. Based on the Heidelberg Bleeding Classification, ICH included hemorrhagic infarction 1, hemorrhagic infarction 2, parenchymal hematoma 1, parenchymal hematoma 2, parenchymal hematoma remote from infarcted brain tissue, intraventricular hemorrhage, subarachnoid hemorrhage, and subdural hemorrhage (10, 21). sICH was defined as ICH accompanied by a relevant neurological deterioration, considered as either an increase in NIHSS score by ≥4 points, an increase by ≥2 points in a NIHSS subcategory, or major medical interventions such as intubation or decompressive craniectomy (15, 21, 22).

### 2.3 Statistical analysis

All statistical analyses and ML model construction were performed using R software version 4.4.2. A *p* < 0.05 was considered statistically significant. In the baseline data, quantitative variables were expressed as medians and interquartile ranges (based on the “quantile” function), and categorical variables were described by counts and proportions (based on the “prop.table” function). Least Absolute Shrinkage and Selection Operator (LASSO) regression was employed to select optimal predictive features (based on the “glmnet” function in the “glmnet” package). Subsequently, incorporating the features, construct a ML model based on multivariate logistic regression (LR) (based on the “glm” function in the “rms” package). Overdispersion test and Variance Inflation Factor (VIF) values assessed model fit, with VIF ≥5 indicating multicollinearity. Goodness-of-fit of the LR model was further evaluated using the Hosmer-Lemeshow test (*p*-value >0.05 indicates adequate model fit). Additionally, a ML model utilizing Support Vector Machine (SVM) was employed to screen for risk factors influencing the outcome (based on the “svm” function in the “e1071” package). The nomogram of the LR model converted multivariate predictors into a visual scoring system (based on the “nomogram” function in the “rms” package). The discriminative performance was evaluated using Receiver Operating Characteristic (ROC) curves and the Area Under the Curve (AUC), and the relatively adjusted AUC calculated through bootstrap validation (1,000 bootstrap resamples) was reported. Calibration of the nomogram was evaluated by calibration curves (1,000 bootstrap resamples).

## 3 Results

**Figure 1:**
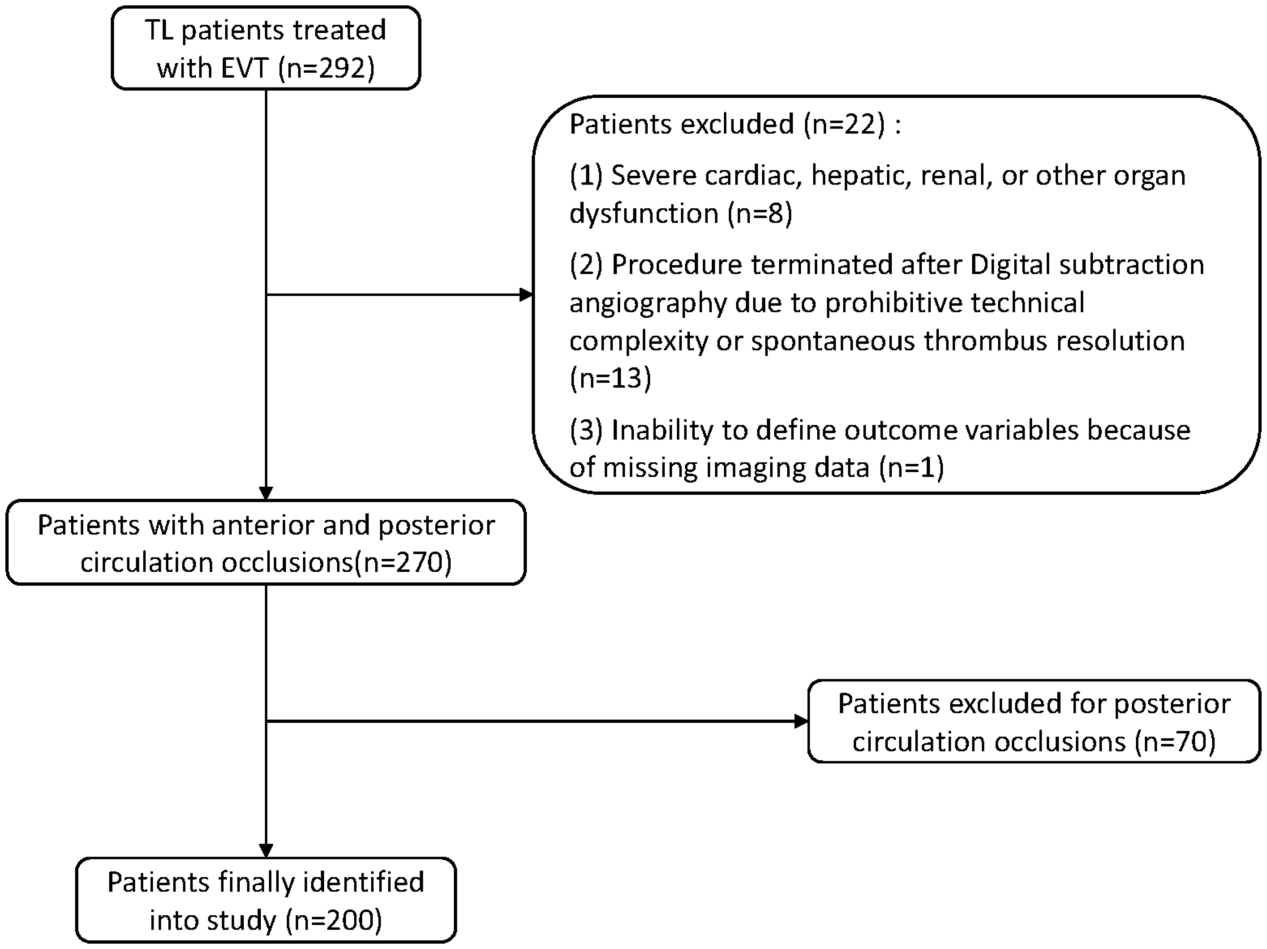
The flowchart of patient selection. TL, tandem lesions; EVT, endovascular thrombectomy.

**Figure 2:**
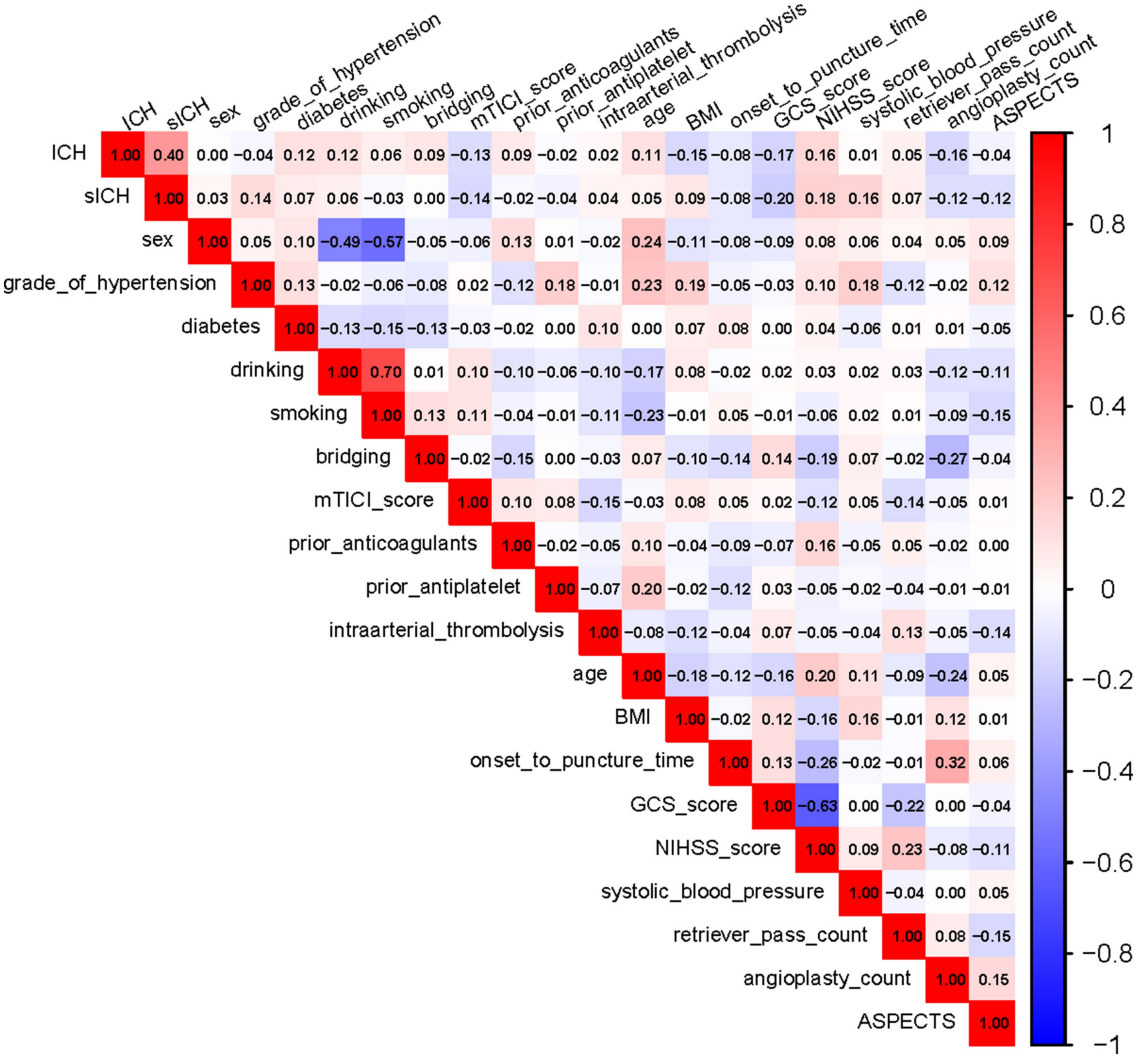
Correlation heatmap of variables. The heatmap depicts the inter-variable correlations. Each square’s value at the intersections represents the correlation coefficient, ranging from −1 (perfect negative correlation) to 1 (perfect positive correlation). Darker squares indicate stronger correlations: red for positive and blue for negative. ICH, intracranial hemorrhage; sICH, symptomatic intracranial hemorrhage; mTICI, modified Thrombolysis in Cerebral Infarction; BMI, body mass index; GCS, Glasgow Coma Scale; NIHSS, National Institutes of Health Stroke Scale; ASPECTS, Alberta Stroke Program Early CT Score.

Based on the inclusion and exclusion criteria, a total of 200 patients with TL involving anterior circulation large vessel occlusion were enrolled in this study between June 2020 and November 2024 (Figure 1). The cohort comprised 141 males (70.5%) and 59 females (29.5%). After EVT, ICH occurred in 92 patients (46%), and sICH occurred in 24 patients (12%). Patient characteristics, including baseline demographics, preoperative clinical data, and procedural details, are presented in Table 1. Correlations between the variables are illustrated in Figure 2.

**Table 1:** Demographic and clinical characteristics of tandem lesions patients.

Characteristic	Overall, *N* = 200	ICH, *N* = 92	No ICH, *N* = 108	sICH, *N* = 24	No sICH, *N* = 176
Sex, n (%)					
Male	141 (70.50%)	65 (70.65%)	76 (70.37%)	16 (66.67%)	125 (71.02%)
Female	59 (29.50%)	27 (29.35%)	32 (29.63%)	8 (33.33%)	51 (28.98%)
Grade of hypertension, n (%)					
0	96 (48.00%)	47 (51.09%)	49 (45.37%)	8 (33.33%)	88 (50.00%)
1	5 (2.50%)	1 (1.09%)	4 (3.70%)	0 (0.00%)	5 (2.84%)
2	15 (7.50%)	6 (6.52%)	9 (8.33%)	1 (4.17%)	14 (7.95%)
3	84 (42.00%)	38 (41.30%)	46 (42.59%)	15 (62.50%)	69 (39.20%)
Diabetes, n (%)					
Yes	42 (21.00%)	24 (26.09%)	18 (16.67%)	7 (29.17%)	35 (19.89%)
No	158 (79.00%)	68 (73.91%)	90 (83.33%)	17 (70.83%)	141 (80.11%)
Drinking, n (%)					
Yes	77 (38.50%)	41 (44.57%)	36 (33.33%)	11 (45.83%)	66 (37.50%)
No	123 (61.50%)	51 (55.43%)	72 (66.67%)	13 (54.17%)	110 (62.50%)
Smoking, n (%)					
Yes	91 (45.50%)	45 (48.91%)	46 (42.59%)	10 (41.67%)	81 (46.02%)
No	109 (54.50%)	47 (51.09%)	62 (57.41%)	14 (58.33%)	95 (53.98%)
Bridging, n (%)					
Yes	116 (58.00%)	58 (63.04%)	58 (53.70%)	14 (58.33%)	102 (57.95%)
No	84 (42.00%)	34 (36.96%)	50 (46.30%)	10 (41.67%)	74 (42.05%)
mTICI score, n (%)					
0	2 (1.00%)	2 (2.17%)	0 (0.00%)	1 (4.17%)	1 (0.57%)
1	5 (2.50%)	4 (4.35%)	1 (0.93%)	1 (4.17%)	4 (2.27%)
2	48 (24.00%)	23 (25.00%)	25 (23.15%)	8 (33.33%)	40 (22.73%)
3	145 (72.50%)	63 (68.48%)	82 (75.92%)	14 (58.33%)	131 (74.43%)
Prior anticoagulants, n (%)					
Yes	11 (5.50%)	7 (7.61%)	4 (3.70%)	1 (4.17%)	10 (5.68%)
No	189 (94.50%)	85 (92.39%)	104 (96.30%)	23 (95.83%)	166 (94.32%)
Prior antiplatelet, n (%)					
Yes	43 (21.50%)	19 (20.65%)	24 (22.22%)	4 (16.67%)	39 (22.16%)
No	157 (78.5%)	73 (79.35%)	84 (77.78%)	20 (83.33%)	137 (77.84%)
Intraarterial thrombolysis, n (%)					
Yes	33 (16.50%)	16 (17.39%)	17 (15.74%)	5 (20.83%)	28 (15.91%)
No	167 (83.50%)	76 (82.61%)	91 (84.26%)	19 (79.17%)	148 (84.09%)
Age, median (IQR)	67 (59, 74)	68.5 (61, 75)	66 (58, 73.25)	67 (60.75, 73.5)	67 (59, 74)
BMI, median (IQR)	24.71 (22.80, 26.35)	24.48 (22.45, 25.70)	25.02 (23.33, 27.34)	24.85 (23.85, 27.23)	24.71 (22.70, 26.25)
Onset to puncture time, (minutes) median (IQR)	302.5 (227.5, 439.25)	303.5 (232.25, 407.75)	302.5 (215.25, 489.5)	278.5 (214.25, 393.5)	304.5 (231.75, 447)
GCS score, median (IQR)	14 (9, 15)	11.5 (8, 15)	14 (10, 15)	9 (8, 14)	14 (9.75, 15)
NIHSS score, median (IQR)	15 (12, 19)	16 (13, 20)	14.5 (12, 18)	18 (15, 22.25)	15 (12, 18.25)
Systolic blood pressure, (mmHg) median (IQR)	149.5 (132, 167)	149.5 (133.5, 164.5)	149.5 (132, 168)	160.5 (144, 183.25)	148 (130.75, 166.25)
Retriever pass count, median (IQR)	1 (1, 2)	1 (1, 2)	1 (1, 1)	1 (0.75, 2)	1 (1, 1)
Angioplasty count, median (IQR)	0 (0, 0)	0 (0, 0)	0 (0, 0)	0 (0, 0)	0 (0, 0)
ASPECTS, median (IQR)	7 (5, 8)	7 (5, 8)	7 (5, 8)	6 (4, 7)	7 (5, 8)

ICH, intracranial hemorrhage; sICH, symptomatic intracranial hemorrhage; mTICI, modified Thrombolysis in Cerebral Infarction; IQR, interquartile range; BMI, body mass index; GCS, Glasgow Coma Scale; NIHSS, National Institutes of Health Stroke Scale; ASPECTS, Alberta Stroke Program Early CT Score.

**Figure 3:**
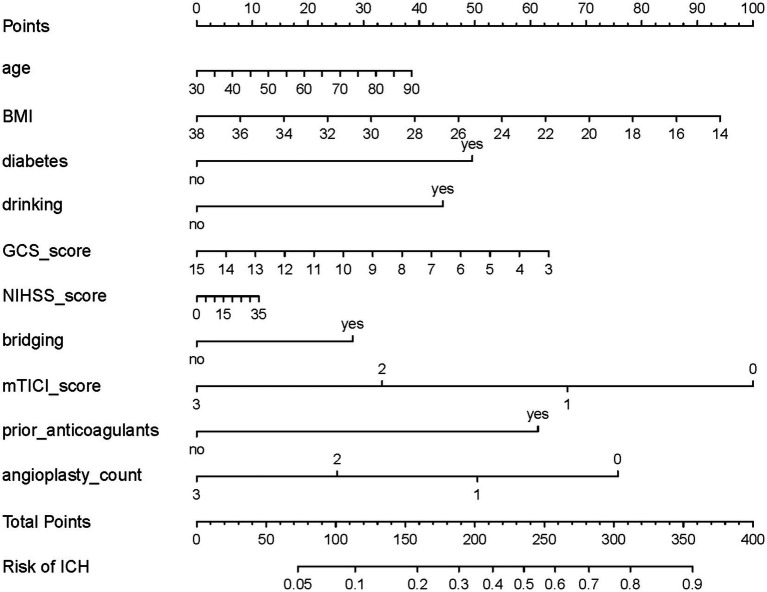
Nomogram for predicting the risk of postoperative ICH in TL patients treated with EVT. To determine the ICH probabilities using the nomogram, locate each variable on its respective axis, project a perpendicular line to the points axis to obtain the corresponding score, sum the individual scores, and then extend a line from the resultant total-points axis downward to intersect the lower probability line. ICH, intracranial hemorrhage; TL, tandem lesions; EVT, endovascular thrombectomy; BMI, body mass index; GCS, Glasgow Coma Scale; NIHSS, National Institutes of Health Stroke Scale; mTICI, modified Thrombolysis in Cerebral Infarction.

**Figure 4:**
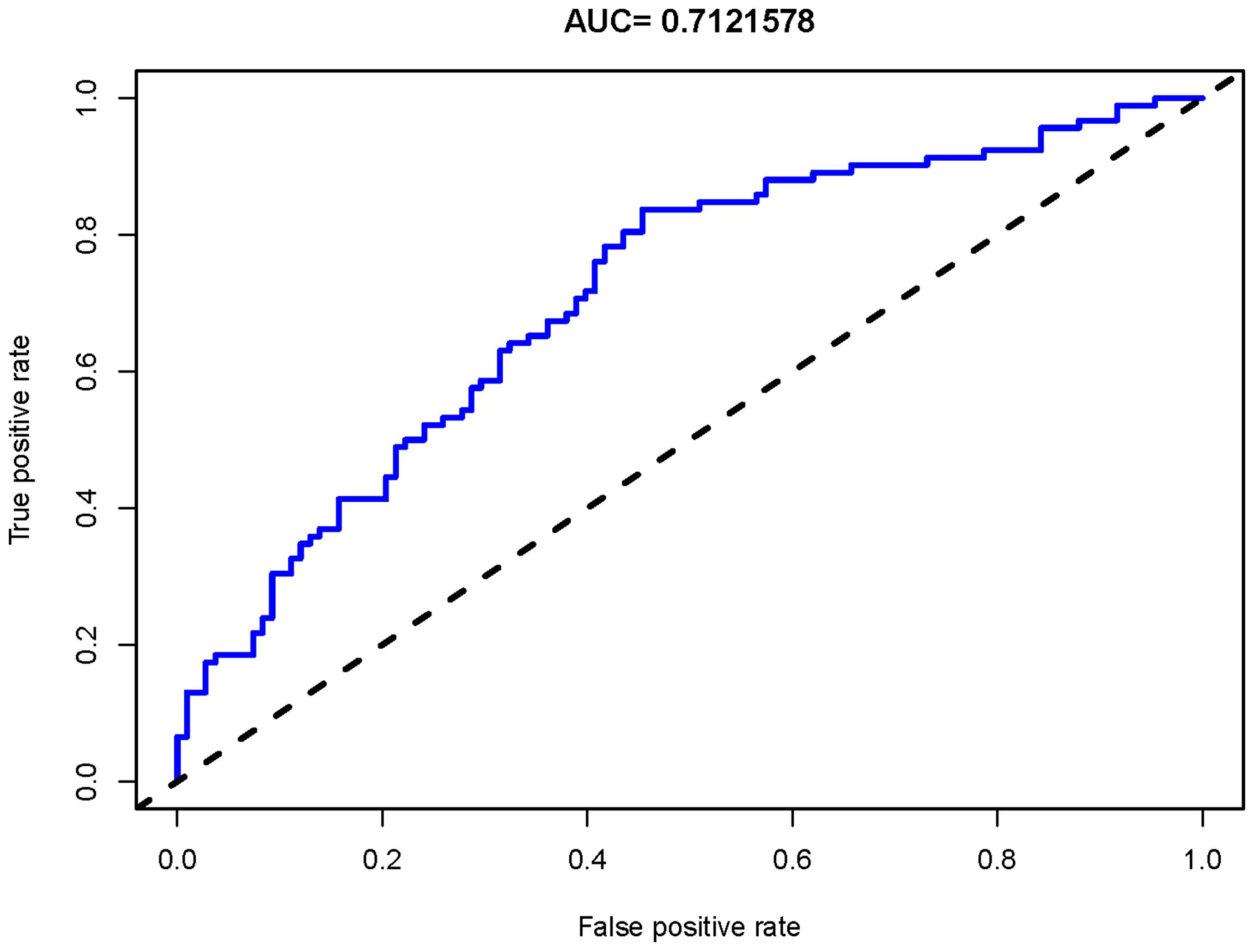
The ROC curve for predicting ICH risk. The curve evaluates the accuracy in predicting ICH risk in TL patients after EVT. It shows the true positive rate against the false positive rate for various thresholds. The dashed line indicates random guessing for comparison. ROC, Receiver Operating Characteristic; ICH, intracranial hemorrhage; TL, tandem lesions; EVT, endovascular thrombectomy; AUC, Area Under the Curve.

LASSO regression was applied to select features influencing ICH after EVT in TL patients, reducing 19 initial features to 10 potential predictors (Supplementary Figures 1, 2). The results of the multivariate LR analysis for these predictors are presented in Table 2. Among them, diabetes, drinking history, and low mTICI score were significantly associated with an increased risk of ICH. Based on the LR model, a nomogram for ICH risk prediction was constructed (Figure 3). The final model demonstrated good fit (overdispersion test: *p* = 0.287) and low variable collinearity (VIF range: 1.077–1.734). The Hosmer-Lemeshow test for the model produced a *p*-value of 0.633. The model exhibited good risk assessment performance (AUC = 0.712, 95% CI: 0.641–0.784), with the ROC curve shown in Figure 4. The adjusted AUC via the bootstrap method was 0.654. Calibration curve and decision curve analysis for the ICH nomogram are presented in Supplementary Figures 3, 4.

**Table 2:** Multivariate logistic regression analysis of postoperative ICH in TL patients.

Variable	*β*	OR (95% CI)	*p*-value
Age	0.012	1.012 (0.981–1.044)	0.465
BMI	−0.071	0.931 (0.845–1.026)	0.152
Diabetes	0.898	2.454 (1.137–5.297)	0.022
Drinking	0.802	2.230 (1.160–4.288)	0.016
GCS score	−0.096	0.909 (0.813–1.016)	0.093
NIHSS score	0.006	1.006 (0.937–1.080)	0.873
Bridging	0.508	1.662 (0.855–3.232)	0.134
mTICI score	−0.604	0.547 (0.311–0.961)	0.036
Prior anticoagulants	1.112	3.040 (0.784–11.783)	0.108
Angioplasty count	−0.457	0.633 (0.335–1.195)	0.158

β is the regression coefficient. ICH, intracranial hemorrhage; TL, tandem lesions; OR, odds ratio; CI, confidence interval; BMI, body mass index; GCS, Glasgow Coma Scale; NIHSS, National Institutes of Health Stroke Scale; mTICI, modified Thrombolysis in Cerebral Infarction.

**Figure 5:**
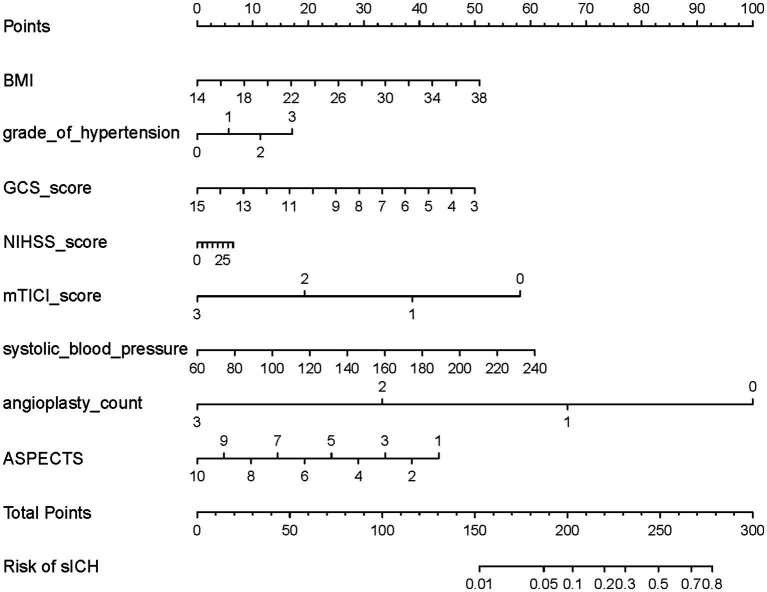
Nomogram for predicting the risk of postoperative sICH in TL patients treated with EVT. To determine the sICH probabilities using the nomogram, locate each variable on its respective axis, project a perpendicular line to the points axis to obtain the corresponding score, sum the individual scores, and then extend a line from the resultant total-points axis downward to intersect the lower probability line. sICH, symptomatic intracranial hemorrhage; TL, tandem lesions; EVT, endovascular thrombectomy; BMI, body mass index; GCS, Glasgow Coma Scale; NIHSS, National Institutes of Health Stroke Scale; mTICI, modified Thrombolysis in Cerebral Infarction; ASPECTS, Alberta Stroke Program Early CT Score.

**Figure 6:**
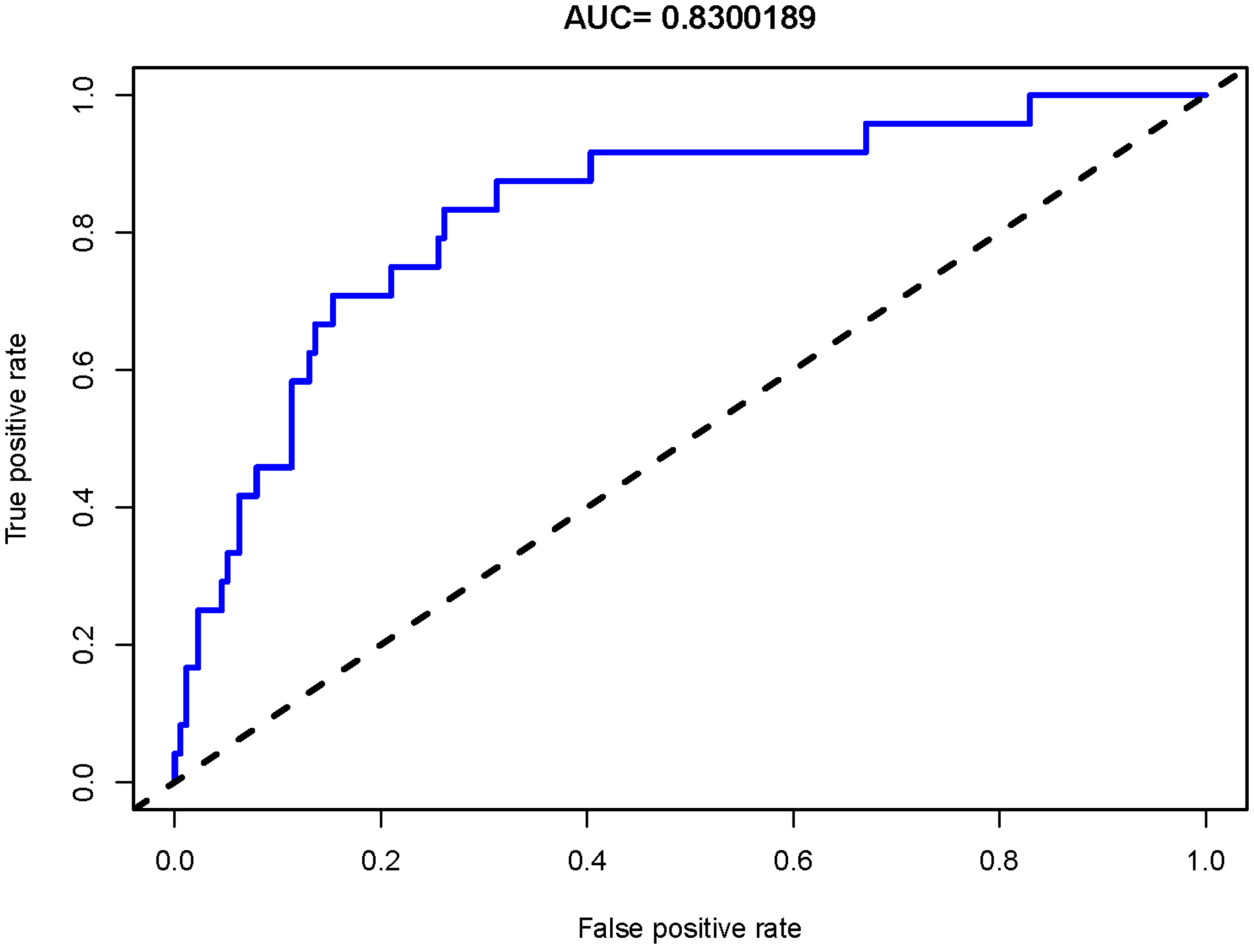
The ROC curve for predicting sICH risk. The curve evaluates the accuracy in predicting sICH risk in TL patients after EVT. It shows the true positive rate against the false positive rate for various thresholds. The dashed line indicates random guessing for comparison. ROC, Receiver Operating Characteristic; sICH, symptomatic intracranial hemorrhage; TL, tandem lesions; EVT, endovascular thrombectomy; AUC, Area Under the Curve.

LASSO regression identified 8 potential predictors influencing sICH after EVT in TL patients (Supplementary Figures 5, 6). The results of the LR analysis for these predictors are presented in Table 3. Among them, low GCS score, low mTICI score, and low ASPECTS were significantly associated with an increased risk of sICH. Based on the LR model, a nomogram for sICH risk prediction was constructed (Figure 5), showing good model fit (overdispersion test: *p* = 0.435) and low collinearity (VIF range: 1.022–1.725). The Hosmer-Lemeshow test for the model produced a *p*-value of 0.638. The model exhibited good risk assessment performance (AUC = 0.830, 95% CI: 0.741–0.919), with the ROC curve shown in Figure 6. The adjusted AUC was 0.773. Calibration curve and decision curve analysis for the sICH nomogram are presented in Supplementary Figures 7, 8.

**Table 3:** Multivariate logistic regression analysis of postoperative sICH in TL patients.

Variable	*β*	OR (95% CI)	*p*-value
BMI	0.101	1.106 (0.952–1.285)	0.189
Grade of hypertension	0.271	1.311 (0.905–1.900)	0.152
GCS score	−0.198	0.820 (0.695–0.968)	0.019
NIHSS score	0.009	1.009 (0.904–1.125)	0.875
mTICI score	−0.922	0.398 (0.182–0.868)	0.021
Systolic blood pressure	0.016	1.016 (0.999–1.034)	0.066
Angioplasty count	−1.587	0.205 (0.030–1.417)	0.108
ASPECTS	−0.230	0.795 (0.641–0.984)	0.035

β is the regression coefficient. sICH, symptomatic intracranial hemorrhage; TL, tandem lesions; OR, odds ratio; CI, confidence interval; BMI, body mass index; GCS, Glasgow Coma Scale; NIHSS, National Institutes of Health Stroke Scale; mTICI, modified Thrombolysis in Cerebral Infarction; ASPECTS, Alberta Stroke Program Early CT Score.

SVM models were constructed using all feature variables. The SVM model predicting ICH risk after EVT in TL patients achieved an AUC of 0.861 (95% CI, 0.812–0.909), and its variable importance plot is shown in Supplementary Figure 9. The SVM model predicting sICH risk achieved an AUC of 0.688 (95% CI, 0.589–0.786), with the variable importance plot presented in Supplementary Figure 10. A summary table of the performance metrics for each model was provided in Supplementary Table 1.

## 4 Discussion

In the LR model predicting hemorrhage risk after EVT in TL patients, diabetes, drinking history, and low mTICI score were significantly associated with an increased risk of ICH; low GCS score, low mTICI score, and low ASPECTS were significantly associated with an increased risk of sICH. The nomogram of the LR model converted multivariate predictors into a visual scoring system, quantifying variable contributions and linking total scores to outcome probabilities. This tool democratizes predictive analytics, supporting real-time, evidence-based decisions during consultations (18, 23, 24).

According to our data characteristics (Table 1), the median baseline ASPECTS for TL patients was seven, lower than previously reported for general AIS populations. The incidence of sICH after EVT in TL patients was 14%, higher than reported in several studies focusing on general AIS patients (15, 18, 22, 25, 26). Furthermore, baseline ASPECTS was a significant risk factor for sICH occurrence. This validates the perspective that patients with TL present with more severe disease and carry a higher risk of sICH complications compared to general AIS patients (3, 7–9). The inherent complexity of TL may compromise reperfusion success, as evidenced by a low mTICI score observed during the procedure (3, 8, 9). Our study also found that low mTICI score was a significant risk factor for both ICH and sICH occurrence (*p* < 0.05). This further suggests that patients with TL may have a higher risk of hemorrhage after EVT (7, 8).

Similar to findings in general AIS populations, diabetes also increased the risk of ICH after EVT in TL patients. Hyperglycemia may contribute to hemorrhage through various biological mechanisms, including impaired cellular metabolism, disruption of vascular integrity, and increased blood–brain barrier (BBB) permeability (7, 16, 20, 22, 27–29). Our study did not find a significant association between hyperglycemia and sICH risk. However, previous studies have shown that hyperglycemia significantly increases the likelihood of sICH after reperfusion therapy and is associated with poor functional outcomes and mortality (16, 25–27). Strict pre-stroke glycemic control (HbA1c ≤ 7.0%) may benefit neurological recovery in patients undergoing EVT (30). Prior research indicates that AIS patients with persistent postoperative hyperglycemia (blood glucose levels >140 mg/dL) have a significantly increased risk of sICH and poor functional outcome after EVT. Another study demonstrated that maintaining fasting blood glucose levels below 11.5 mmol/L on postoperative day 1 reduces the incidence of poor prognosis (31, 32). Therefore, for diabetic patients with TL, early identification and control of perioperative blood glucose levels are crucial for improving outcomes after EVT.

This study proposes a novel finding that drinking history may be an independent risk factor for ICH after EVT in TL patients. Previous research in stroke patients has shown that excess alcohol consumption is an independent risk factor for spontaneous hemorrhagic transformation (33). A study in rats has indicated that blood alcohol concentrations >300 mg/dL can induce irreversible spasm and even rupture with hemorrhage in the cortical microvessels (34). Alcohol may promote hemorrhagic events through mechanisms such as elevated blood pressure, vascular wall damage, exacerbation of neuroinflammation, and BBB disruption (20, 29, 35, 36).

Previous research has established that in AIS patients undergoing EVT, the mTICI score correlates with both ICH and sICH, with low mTICI score identified as an independent predictor of hemorrhage (15, 25). Low mTICI score may exacerbate injury in the infarcted area, increase the risk of hemorrhagic transformation, and facilitate it by damaging the BBB and increasing vascular permeability (20, 29, 37). Operator caution, potentially driven by concerns that frequent device manipulation (e.g., multiple stent retriever passes) could disrupt the BBB, might contribute to suboptimal reperfusion (7, 15, 20, 22, 26, 28). However, this study did not find that procedural factors like the number of stent retriever passes or intraarterial thrombolysis significantly influenced hemorrhage risk. This suggests that proactive surgical intervention during EVT for TL patients may reduce postoperative hemorrhage risk and benefit patients.

In contrast to some prior studies, low GCS score emerged as a significant predictor of sICH after EVT in TL patients. Although NIHSS score was not significant in the multivariate LR (Table 3), it was retained in the nomogram (Figure 5) after selection by LASSO regression. NIHSS score can complement GCS in predicting outcomes and identifying hemorrhagic transformation (26, 38, 39). The lack of significance for NIHSS in the LR analysis might be related to its strong negative correlation with GCS score (Figure 2). Patients with low GCS scores often present with high NIHSS scores, and this high correlation may have suppressed the independent effect of NIHSS in the multivariate model, preventing it from reaching statistical significance (23, 38, 40). Therefore, while GCS demonstrates a robust association with poor outcomes, incorporating variables such as NIHSS from the nomogram into a comprehensive analysis enhances the reliability of clinical decision-making.

Similar to findings in general AIS populations, this study found that low ASPECTS also increases the risk of sICH after EVT in TL patients (16, 17, 22, 28). ASPECTS serves as a surrogate marker for infarct volume; a lower ASPECTS value indicates greater irreversible ischemic brain tissue damage and is associated with an increased risk of hemorrhagic transformation through mechanisms involving vascular injury, BBB disruption, and inflammatory responses (16, 20, 29, 41). A multicenter retrospective study found that the potential benefit of functional independence following EVT in some TL patients with ASPECTS of 0–5 was non-negligible (17). Likely due to this phenomenon, operators at our center, after thorough informed consent, did not decline EVT for these patients. This underscores the clinical relevance of our study: when encountering a single high-risk factor for sICH, a comprehensive assessment incorporating other factors from the nomogram enables personalized prognosis evaluation, guiding clinical decision-making.

The application of ML algorithms for variable selection and nomogram construction to predict hemorrhage risk after EVT in TL patients represents a novel approach. In the variable importance plots of the SVM models, the top nine variables associated with ICH and the top seven variables associated with sICH all belonged to the key variables identified via LASSO regression (Supplementary Figures 9, 10), further validating the reliability of the LASSO variable selection. The constructed nomograms demonstrated good discriminative ability, providing TL patients with relatively accurate predictive tools for assessing postoperative ICH risk.

We acknowledge several limitations in this study. Firstly, the study was retrospective. Despite the implementation of strict inclusion and exclusion criteria, it remained challenging to entirely eliminate biases among the outcomes. Due to the retrospective nature of the data, imaging data such as precise infarct core volume and relative cerebral blood flow ratios were unavailable; we utilized ASPECTS as a surrogate marker for infarct volume (16, 20). Additionally, we did not perform dynamic monitoring of perioperative blood pressure and blood glucose levels in TL patients (31, 32, 42). Secondly, the model was developed using data derived from Chinese patients. Its applicability to populations in other countries has not been validated, necessitating enhanced collaboration with other international stroke centers to improve the model’s generalizability (25, 43). Thirdly, although the creation of predictive models demands “big data,” there is currently no standardized criteria to determine an appropriate sample size. Increasing the sample size or applying data balancing techniques in the future may enhance the model’s predictive performance (18, 26, 44). Despite these limitations, the final predictive model from this study exhibited good performance and did not appear to be adversely affected by them.

## 5 Conclusion

This study demonstrates that in AIS patients with anterior circulation TL, diabetes, drinking history, and low mTICI score significantly increase the risk of ICH following EVT. Meanwhile, low GCS score, low mTICI score, and low ASPECTS significantly increase the risk of sICH. Furthermore, the nomograms constructed using ML models in this study quantify the contribution of variables and link total scores to outcome probabilities, thereby assisting clinicians in rapidly assessing hemorrhage risk for personalized prognosis evaluation and treatment guidance.

## Data Availability

The raw data supporting the conclusions of this article will be made available by the authors, without undue reservation.

## References

[1] Fan J Li X Yu X Liu Z Jiang Y Fang Y. Global burden, risk factors analysis, and prediction study of ischemic stroke, 1990-2030. Neurology. (2023) 101:e137–50. doi: 10.1212/WNL.0000000000207387, PMID: 37197995 PMC10351546

[2] Phipps MS Cronin CA. Management of acute ischemic stroke. BMJ. (2020) 368:l6983. doi: 10.1136/bmj.l6983, PMID: 32054610

[3] Kim YS Garami Z Mikulik R Molina CA Alexandrov AV. Early recanalization rates and clinical outcomes in patients with tandem internal carotid artery/middle cerebral artery occlusion and isolated middle cerebral artery occlusion. Stroke. (2005) 36:869–71. doi: 10.1161/01.STR.0000160007.57787.4c, PMID: 15746449

[4] D’Anna L Foschi M Valente M Zhang L Sacco S Ornello R. Impact of sex on clinical outcomes of tandem occlusion in acute ischemic stroke patients treated with mechanical thrombectomy. A propensity-matched analysis. Eur J Neurol. (2025) 32:e70044. doi: 10.1111/ene.70044, PMID: 39804012 PMC11726627

[5] Hernández-Fernández F Del Valle Pérez JA García-García J Ayo-Martín Ó Ramos-Araque ME Molina-Nuevo JD. Simultaneous angioplasty and mechanical Thrombectomy in tandem carotid occlusions. Incidence of reocclusions and prognostic predictors. J Stroke Cerebrovasc Dis. (2020) 29:104578. doi: 10.1016/j.jstrokecerebrovasdis.2019.104578, PMID: 31866200

[6] Zhu F Labreuche J Haussen D Piotin M Steglich-Arnholm H Taschner C. Hemorrhagic transformation after thrombectomy for tandem occlusions: incidence, predictors, and clinical implications. Stroke. (2019) 50:516–9. doi: 10.1161/STROKEAHA.118.023689, PMID: 30580731

[7] Vora N Gupta R Thomas A Horowitz M Tayal A Hammer M. Factors predicting hemorrhagic complications after multimodal reperfusion therapy for acute ischemic stroke. Am J Neuroradiol. (2007) 28:1391–4. doi: 10.3174/ajnr.A0575, PMID: 17698549 PMC7977651

[8] Nolan NM Regenhardt RW Koch MJ Raymond SB Stapleton CJ Rabinov JD. Treatment approaches and outcomes for acute anterior circulation stroke patients with tandem lesions. J Stroke Cerebrovasc Dis. (2021) 30:105478. doi: 10.1016/j.jstrokecerebrovasdis.2020.105478, PMID: 33248344 PMC7855424

[9] Poppe A Jacquin G Roy D Stapf C Derex L. Tandem carotid lesions in acute ischemic stroke: mechanisms, therapeutic challenges, and future directions. Am J Neuroradiol. (2020) 41:1142–8. doi: 10.3174/ajnr.A6582, PMID: 32499251 PMC7357657

[10] Fekete KE Héja M Márton S Tóth J Harman A Horváth L. Predictors and long-term outcome of intracranial hemorrhage after thrombolytic therapy for acute ischemic stroke—a prospective single-center study. Front Neurol. (2023) 14:1080046. doi: 10.3389/fneur.2023.1080046, PMID: 36816554 PMC9929139

[11] Heit J Mlynash M Christensen S Kemp S Lansberg M Marks M. What predicts poor outcome after successful thrombectomy in late time windows? J Neurointerv Surg. (2020) 13:421–5. doi: 10.1136/neurintsurg-2020-016125, PMID: 32554693

[12] Renú A Amaro S Laredo C Román LS Llull L Lopez A. Relevance of blood–brain barrier disruption after endovascular treatment of ischemic stroke. Stroke. (2015) 46:673–9. doi: 10.1161/STROKEAHA.114.008147, PMID: 25657188

[13] Zhu F Anadani M Labreuche J Spiotta A Turjman F Piotin M. Impact of antiplatelet therapy during endovascular therapy for tandem occlusions: a collaborative pooled analysis. Stroke. (2020) 51:1522–9. doi: 10.1161/STROKEAHA.119.028231, PMID: 32188367

[14] Liu K Jiang L Ruan J Xia W Huang H Niu G. The role of dual energy CT in evaluating hemorrhagic complications at different stages after Thrombectomy. Front Neurol. (2020) 11:583411. doi: 10.3389/fneur.2020.583411, PMID: 33117268 PMC7575741

[15] Ringleb P Schieber S Möhlenbruch M Neuberger U Bendszus M Kickingereder P. Risk factors of intracranial hemorrhage after mechanical thrombectomy of anterior circulation ischemic stroke. Neuroradiology. (2019) 61:461–9. doi: 10.1007/s00234-019-02180-6, PMID: 30778621

[16] Kuang Y Zhang L Ye K Jiang Z Shi C Luo L. Clinical and imaging predictors for hemorrhagic transformation of acute ischemic stroke after endovascular thrombectomy. J Neuroimaging. (2024) 34:339–47. doi: 10.1111/jon.13191, PMID: 38296794

[17] Galecio-Castillo M Farooqui M Guerrero WR Ribo M Hassan AE Jumaa MA. Endovascular treatment of patients with acute ischemic stroke with tandem lesions presenting with low Alberta stroke program early computed tomography score. J Am Heart Assoc. (2024) 13:e035977. doi: 10.1161/JAHA.124.035977, PMID: 39508172 PMC11681390

[18] Duan Q Li W Zhang Y Zhuang W Long J Wu B. Nomogram established on account of Lasso-logistic regression for predicting hemorrhagic transformation in patients with acute ischemic stroke after endovascular thrombectomy. Clin Neurol Neurosurg. (2024) 243:108389. doi: 10.1016/j.clineuro.2024.108389, PMID: 38870670

[19] Payabvash S Qureshi MH Khan SM Khan M Majidi S Pawar S. Differentiating intraparenchymal hemorrhage from contrast extravasation on post-procedural noncontrast CT scan in acute ischemic stroke patients undergoing endovascular treatment. Neuroradiology. (2014) 56:737–44. doi: 10.1007/s00234-014-1381-8, PMID: 24925217

[20] Hoche C Henderson A Ifergan H Gaudron M Magni C Maldonado I. Determinants and clinical relevance of iodine contrast extravasation after endovascular Thrombectomy: a dual-energy CT study. Am J Neuroradiol. (2023) 45:30–6. doi: 10.3174/ajnr.A8081, PMID: 38323978 PMC10756568

[21] von Kummer R Broderick JP Campbell BC Demchuk A Goyal M Hill MD. The Heidelberg bleeding classification: classification of bleeding events after ischemic stroke and reperfusion therapy. Stroke. (2015) 46:2981–6. doi: 10.1161/STROKEAHA.115.010049, PMID: 26330447

[22] Zhang X Xie Y Wang H Yang D Jiang T Yuan K. Symptomatic intracranial hemorrhage after mechanical Thrombectomy in Chinese ischemic stroke patients: the ASIAN score. Stroke. (2020) 51:2690–6. doi: 10.1161/STROKEAHA.120.030173, PMID: 32811387

[23] Stoltzfus J. Logistic regression: a brief primer. Acad Emerg Med Off J Soc Acad Emerg Med. (2011) 18:1099–104. doi: 10.1111/j.1553-2712.2011.01185.x, PMID: 21996075

[24] Shariat S Capitanio U Jeldres C Karakiewicz P. Can nomograms be superior to other prediction tools? BJU Int. (2009) 103:492–7. doi: 10.1111/j.1464-410X.2008.08073.x, PMID: 18990135

[25] Tang C-W Lin K Liu H-M Wei C-Y Chiou H Chou C-L. Predictive modeling of symptomatic intracranial hemorrhage following endovascular Thrombectomy: insights from the Nationwide TREAT-AIS registry. J Stroke. (2025) 27:85–94. doi: 10.5853/jos.2024.04119, PMID: 39916457 PMC11834349

[26] Tian B Tian X Shi Z Peng W Zhang X Yang P. Clinical and imaging indicators of hemorrhagic transformation in acute ischemic stroke after endovascular Thrombectomy. Stroke. (2021) 53:1674–81. doi: 10.1161/STROKEAHA.121.035425, PMID: 34872341

[27] Bradley SA Smokovski I Bhaskar SMM. Impact of diabetes on clinical and safety outcomes in acute ischemic stroke patients receiving reperfusion therapy: a meta-analysis. Adv Clin Exp Med. (2022) 31:583–96. doi: 10.17219/acem/146273, PMID: 35212489 PMC13539098

[28] Sun J Lam C Christie L Blair C Li X Werdiger F. Risk factors of hemorrhagic transformation in acute ischaemic stroke: a systematic review and meta-analysis. Front Neurol. (2023) 14:1079205. doi: 10.3389/fneur.2023.1079205, PMID: 36891475 PMC9986457

[29] Szepanowski RD Haupeltshofer S Vonhof SE Frank B Kleinschnitz C Casas AI. Thromboinflammatory challenges in stroke pathophysiology. Semin Immunopathol. (2023) 45:389–410. doi: 10.1007/s00281-023-00994-4, PMID: 37273022 PMC10241149

[30] Chang J Kim W-J Kwon J Kim B Kim J Lee J. Prestroke glucose control and functional outcome in patients with acute large vessel occlusive stroke and diabetes after thrombectomy. Diabetes Care. (2021) 44:2140–8. doi: 10.2337/dc21-0271, PMID: 34215632 PMC8740925

[31] Merlino G Smeralda C Sponza M Gigli G Lorenzut S Marini A. Dynamic hyperglycemic patterns predict adverse outcomes in patients with acute ischemic stroke undergoing mechanical thrombectomy. Journal of. Clin Med. (2020) 9:9. doi: 10.3390/jcm9061932, PMID: 32575739 PMC7355777

[32] Wang Y Ren H Lin XY Zhang X Luo B. The impact of blood glucose levels on the prognosis of diabetic patients with successful recanalization after mechanical thrombectomy: a single-center retrospective study. J Craniofac Surg. (2024) 35:e740–3. doi: 10.1097/SCS.0000000000010549, PMID: 39320098

[33] Cao Y Wang C Wang Y Wang A Chen G Zheng H. Frequency and risk factors of spontaneous hemorrhagic transformation following ischemic stroke on the initial brain CT or MRI: data from the China National Stroke Registry (CNSR). Neurol Res. (2016) 38:538–44. doi: 10.1080/01616412.2016.1187864, PMID: 27320249

[34] Altura B Altura B Gebrewold A. Alcohol-induced spasms of cerebral blood vessels: relation to cerebrovascular accidents and sudden death. Science. (1983) 220:331–3. doi: 10.1126/science.6836278, PMID: 6836278

[35] Wei J Dai Y Wen W Li J Ye LL Xu S. Blood-brain barrier integrity is the primary target of alcohol abuse. Chem Biol Interact. (2021) 337:109400. doi: 10.1016/j.cbi.2021.109400, PMID: 33516661

[36] Sriram U Winfield M Grove D Mogadala N Rom S Mekala N. Neuroinflammatory responses and blood–brain barrier injury in chronic alcohol exposure: role of purinergic P2 × 7 receptor signaling. J Neuroinflammation. (2024) 21:244. doi: 10.1186/s12974-024-03230-4, PMID: 39342243 PMC11439317

[37] Neuberger U Ringleb P Ulfert C Heiland S Brugnara G Hansen MB. Dynamics of cerebral perfusion and oxygenation parameters following endovascular treatment of acute ischemic stroke. Journal of Neurointerventional. Surgery. (2021) 14:neurintsurg-2020-017163. doi: 10.1136/neurintsurg-2020-017163, PMID: 33762405 PMC8785045

[38] Dusenbury W Tsivgoulis G Chang J Goyal N Swatzell V Alexandrov A. Validation of the National Institutes of Health stroke scale in intracerebral hemorrhage. Stroke: vascular and interventional. Neurology. (2023) 3:e000834. doi: 10.1161/SVIN.123.000834, PMID: 40751573

[39] Li Q-X Fan H Wang D-L Li X-N Wang X-J Zhang L. Application values of six scoring Systems in the Prognosis of stroke patients. Front Neurol. (2020) 10:1416. doi: 10.3389/fneur.2019.01416, PMID: 32082237 PMC7002556

[40] Baby P Pr S Reddy AV Rajasekaran AK Philip M Akkunje PS. Transcultural adaptation and validation of Kannada version of the National Institute of health stroke scale (NIHSS). Annals of Indian academy of. Neurology. (2022) 25:224–8. doi: 10.4103/aian.aian_707_21 PMC9175408 35693651

[41] Nawar E Yeung J Labreuche J Chadenat M Duong D De Malherbe M. MRI-based predictors of hemorrhagic transformation in patients with stroke treated by intravenous thrombolysis. Front Neurol. (2019) 10:897. doi: 10.3389/fneur.2019.00897, PMID: 31507511 PMC6719609

[42] Silverman A Kodali S Sheth KN Petersen NH. Hemodynamics and hemorrhagic transformation after endovascular therapy for ischemic stroke. Front Neurol. (2020) 11:728. doi: 10.3389/fneur.2020.00728, PMID: 32765416 PMC7379334

[43] Ho SY Phua K Wong L Bin Goh WW. Extensions of the external validation for checking learned model interpretability and generalizability. Patterns. (2020) 1:100129. doi: 10.1016/j.patter.2020.100129, PMID: 33294870 PMC7691387

[44] Riley RD Ensor J Snell KIE Harrell FE Jr Martin GP Reitsma JB. Calculating the sample size required for developing a clinical prediction model. BMJ. (2020) 368:m441. doi: 10.1136/bmj.m441, PMID: 32188600

