# Supplementary material for: Intracranial hemorrhage prediction in acute ischemic stroke patients with anterior circulation tandem lesions following endovascular thrombectomy
Source: Front Neurol. 2025 Aug 29;16:1598203. doi: 10.3389/fneur.2025.1598203 (PMC12426950; doi:10.3389/fneur.2025.1598203)
Supplement: Supplementary file 1 [file Data_Sheet_1.docx]

Supplementary Material

**
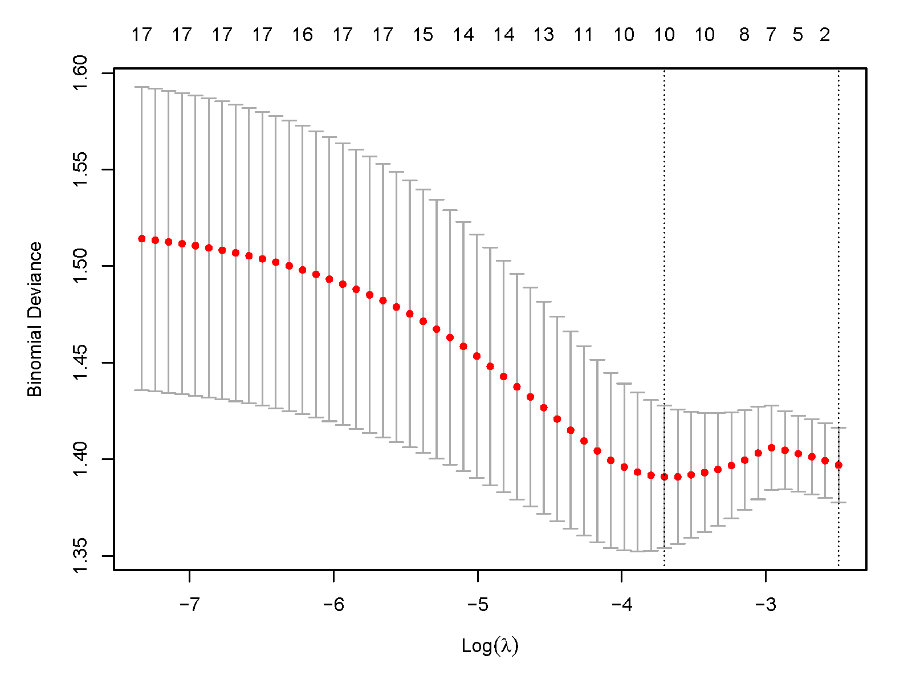
**

**Supplementary Figure 1.** The relationship plot between the partial likelihood deviance (binomial deviance) curve and log(lambda) for predicting ICH.

In the LASSO model, the optimal parameter (lambda) is selected via 10-fold cross-validation using the minimum criterion. Dashed vertical lines are drawn at the minimum criterion and the 1-SE criterion. ICH, intracranial hemorrhage; LASSO, Least Absolute Shrinkage and Selection Operator; SE, standard error.

**
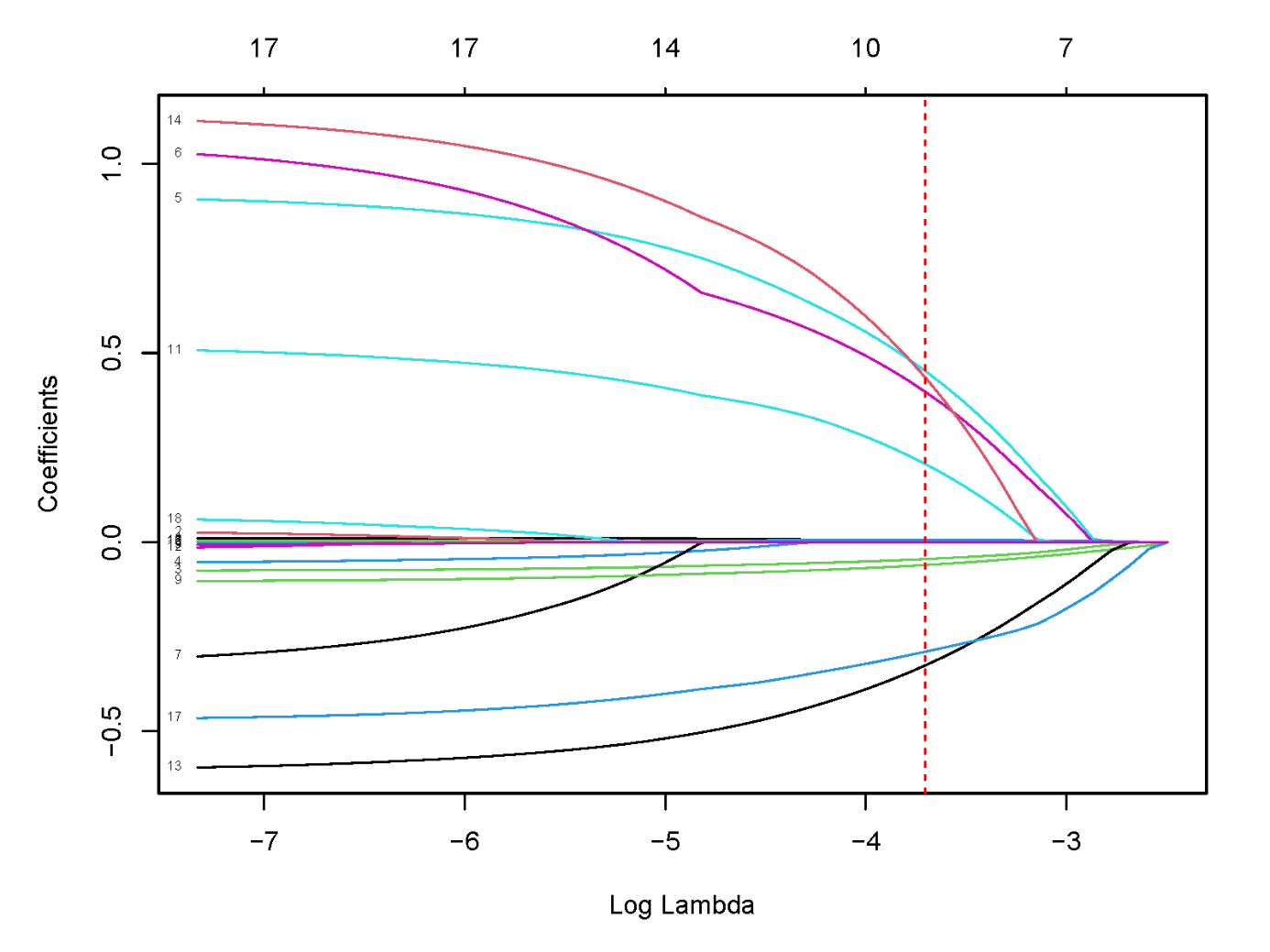
**

**Supplementary Figure 2.** The LASSO coefficient profile plot for predicting ICH.

The coefficient profiles are generated for a sequence of log(lambda) values, with a vertical line drawn at the value selected by 10-fold cross-validation. At this optimal lambda, ten features have non-zero coefficients. LASSO, Least Absolute Shrinkage and Selection Operator; ICH, intracranial hemorrhage.

**
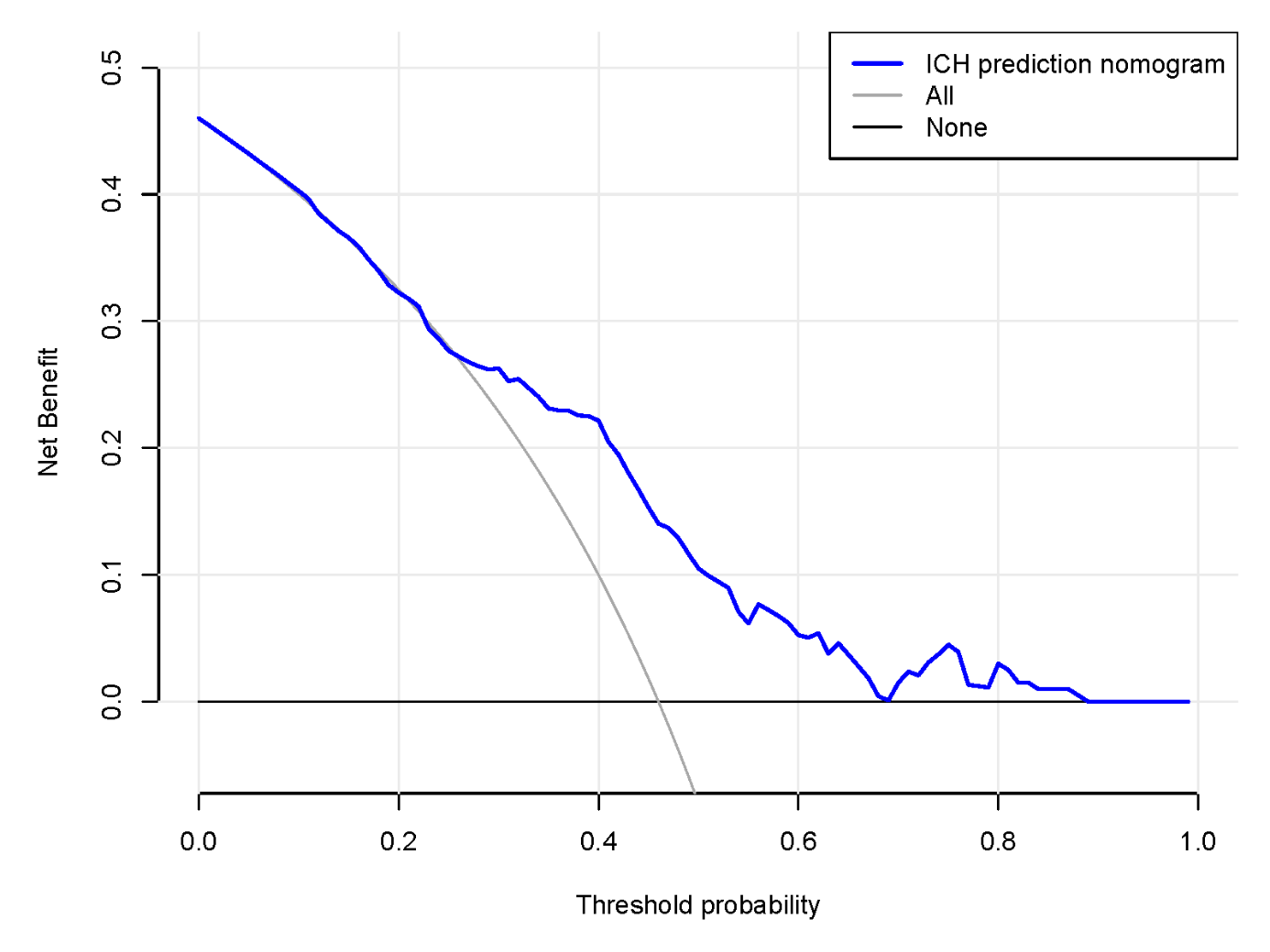
**

**Supplementary Figure 3**. Decision curve analysis of the ICH risk nomogram.

The blue line corresponds to the ICH risk nomogram. The thin solid line signifies the scenario where all patients are assumed to experience ICH, while the thick solid line represents the scenario where no patients are assumed to experience ICH. The decision curve confirmed clinical utility across thresholds of 0.04-0.89. ICH, intracranial hemorrhage.

**

**

**Supplementary Figure 4.** Calibration curve of the ICH risk nomogram.

The dashed diagonal line denotes the line of perfect discrimination for an ideal model, whereas the solid line depicts the observed performance of the nomogram; closer alignment of the solid line with the dashed diagonal indicates superior prediction. ICH, intracranial hemorrhage.

**
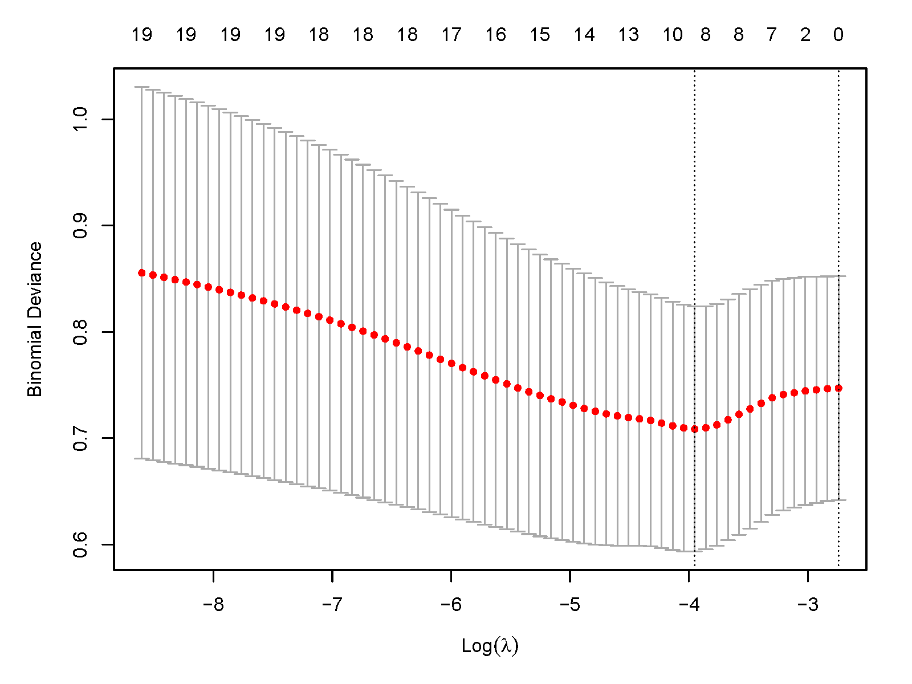
**

**Supplementary Figure 5.** The relationship plot between the partial likelihood deviance (binomial deviance) curve and log(lambda) for predicting sICH.

In the LASSO model, the optimal parameter (lambda) is selected via 10-fold cross-validation using the minimum criterion. Dashed vertical lines are drawn at the minimum criterion and the 1-SE criterion. sICH, symptomatic intracranial hemorrhage; LASSO, Least Absolute Shrinkage and Selection Operator; SE, standard error.

**
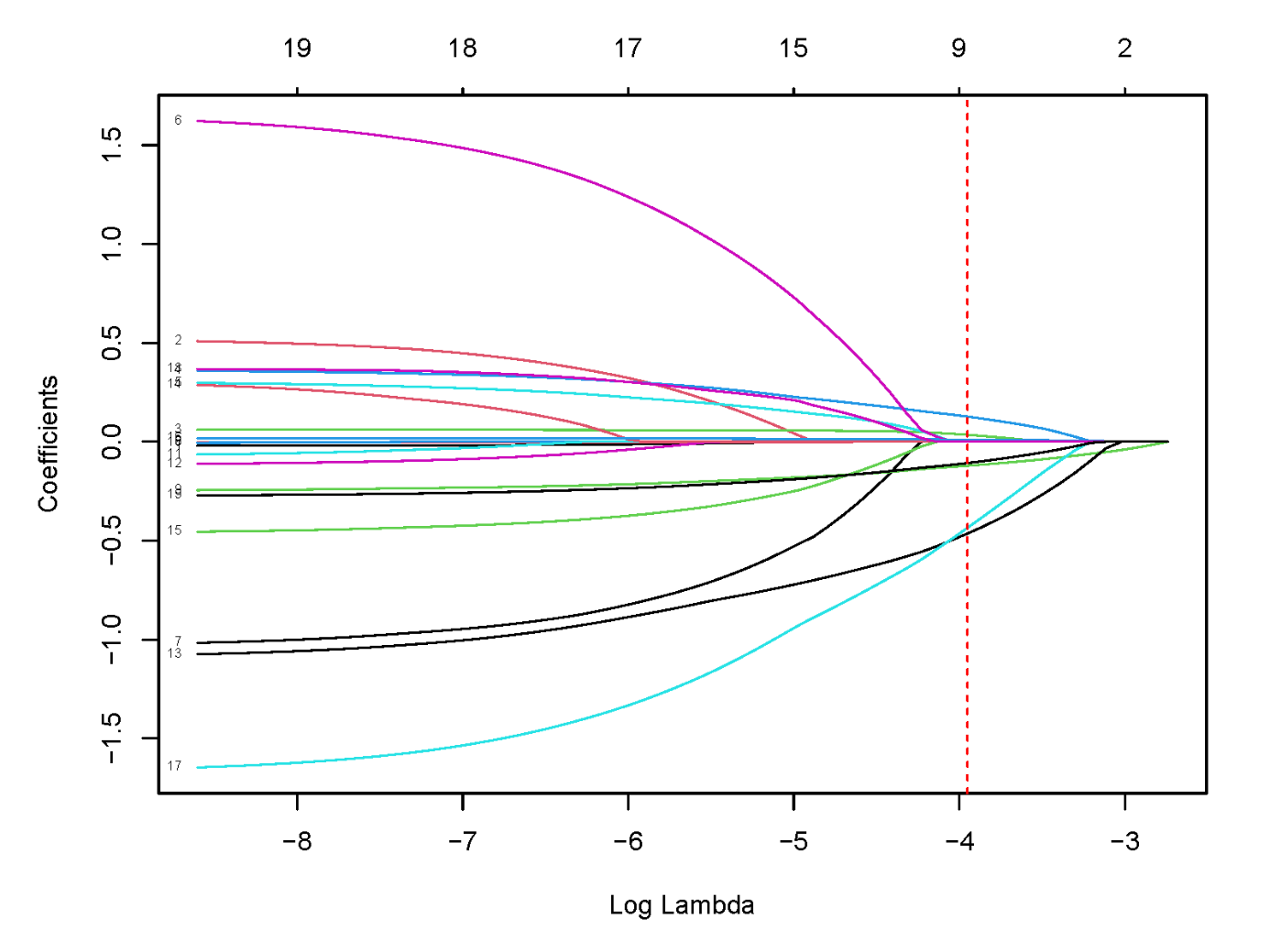
**

**Supplementary Figure 6.** The LASSO coefficient profile plot for predicting sICH.

The coefficient profiles are generated for a sequence of log(lambda) values, with a vertical line drawn at the value selected by 10-fold cross-validation. At this optimal lambda, eight features have non-zero coefficients. LASSO, Least Absolute Shrinkage and Selection Operator; sICH, symptomatic intracranial hemorrhage.

**
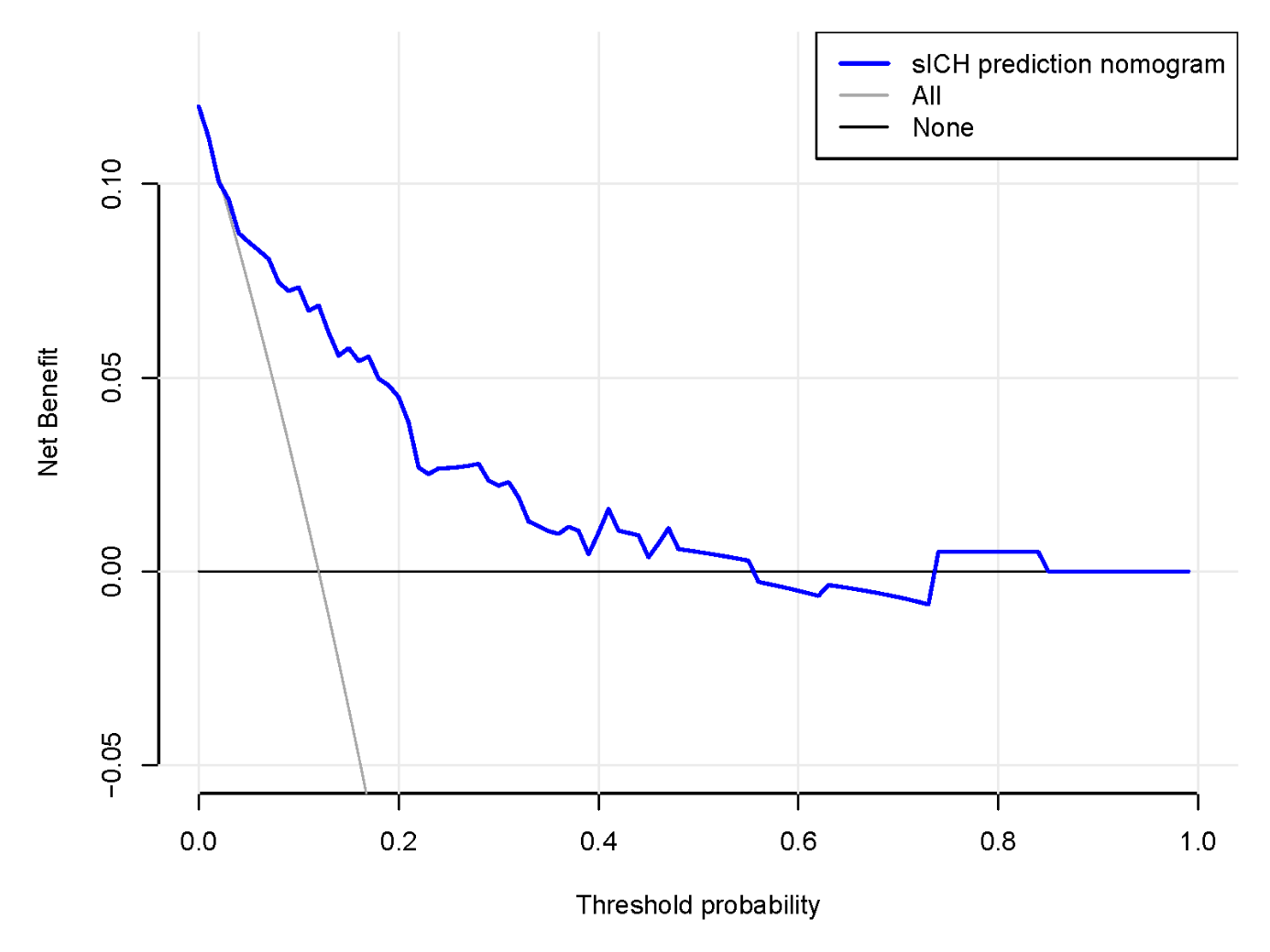
**

**Supplementary Figure 7**. Decision curve analysis of the sICH risk nomogram.

The blue line corresponds to the ICH risk nomogram. The thin solid line signifies the scenario where all patients are assumed to experience sICH, while the thick solid line represents the scenario where no patients are assumed to experience sICH. The decision curve confirmed clinical utility across thresholds of 0.01-0.55 and 0.74-0.84. sICH, symptomatic intracranial hemorrhage.

**

**

**Supplementary Figure 8.** Calibration curve of the sICH risk nomogram.

The dashed diagonal line denotes the line of perfect discrimination for an ideal model, whereas the solid line depicts the observed performance of the nomogram; closer alignment of the solid line with the dashed diagonal indicates superior prediction. sICH, symptomatic intracranial hemorrhage.

**
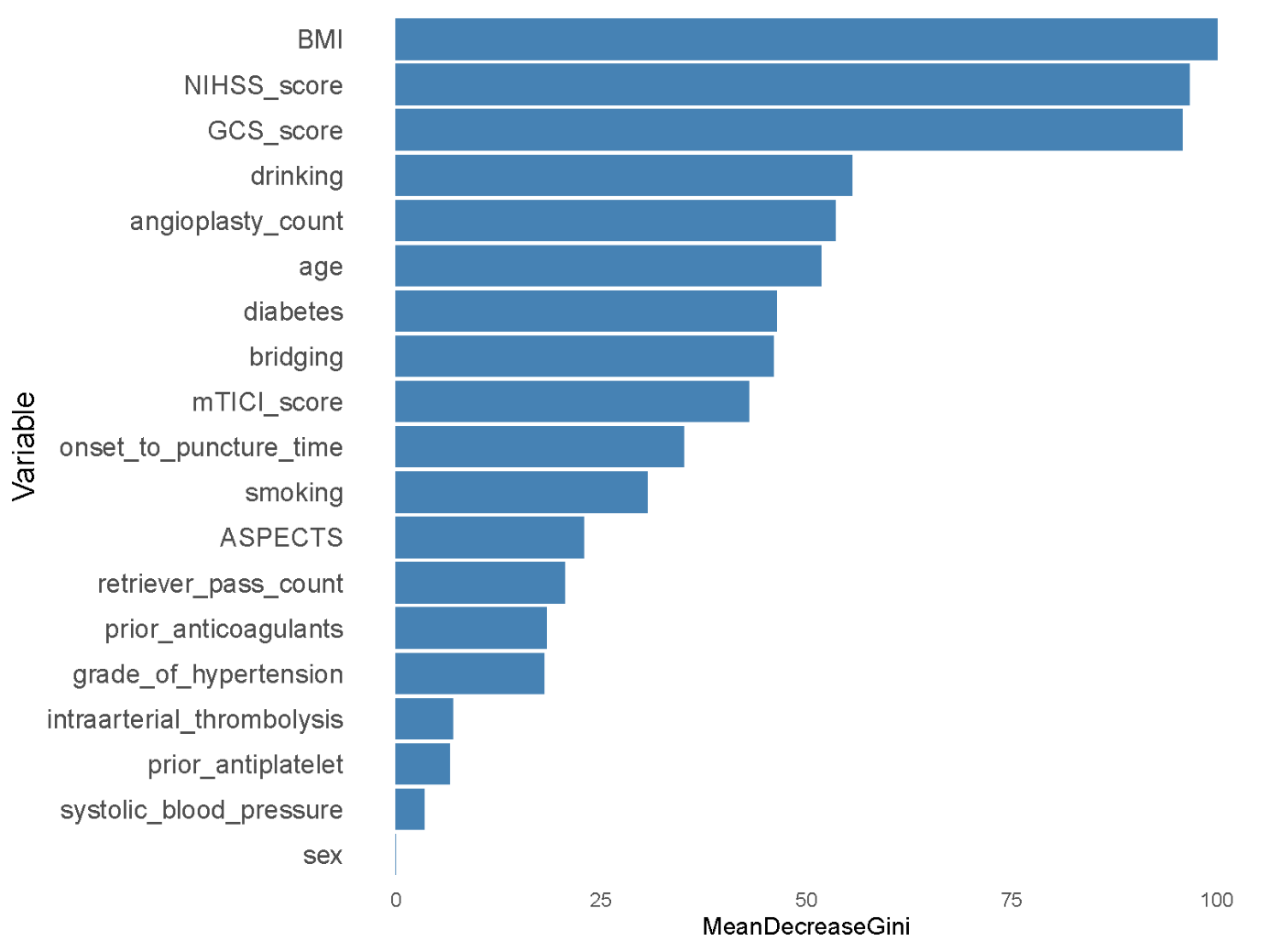
**

**Supplementary Figure 9.** Variable importance plot for the SVM model predicting ICH.

Each bar in the figure represents a feature, with the length of the bar indicating the importance value of that feature. SVM, Support Vector Machine; ICH, intracranial hemorrhage; mTICI, modified Thrombolysis in Cerebral Infarction; BMI, body mass index; GCS, Glasgow Coma Scale; NIHSS, National Institutes of Health Stroke Scale; ASPECTS, Alberta Stroke Program Early CT Score.

**
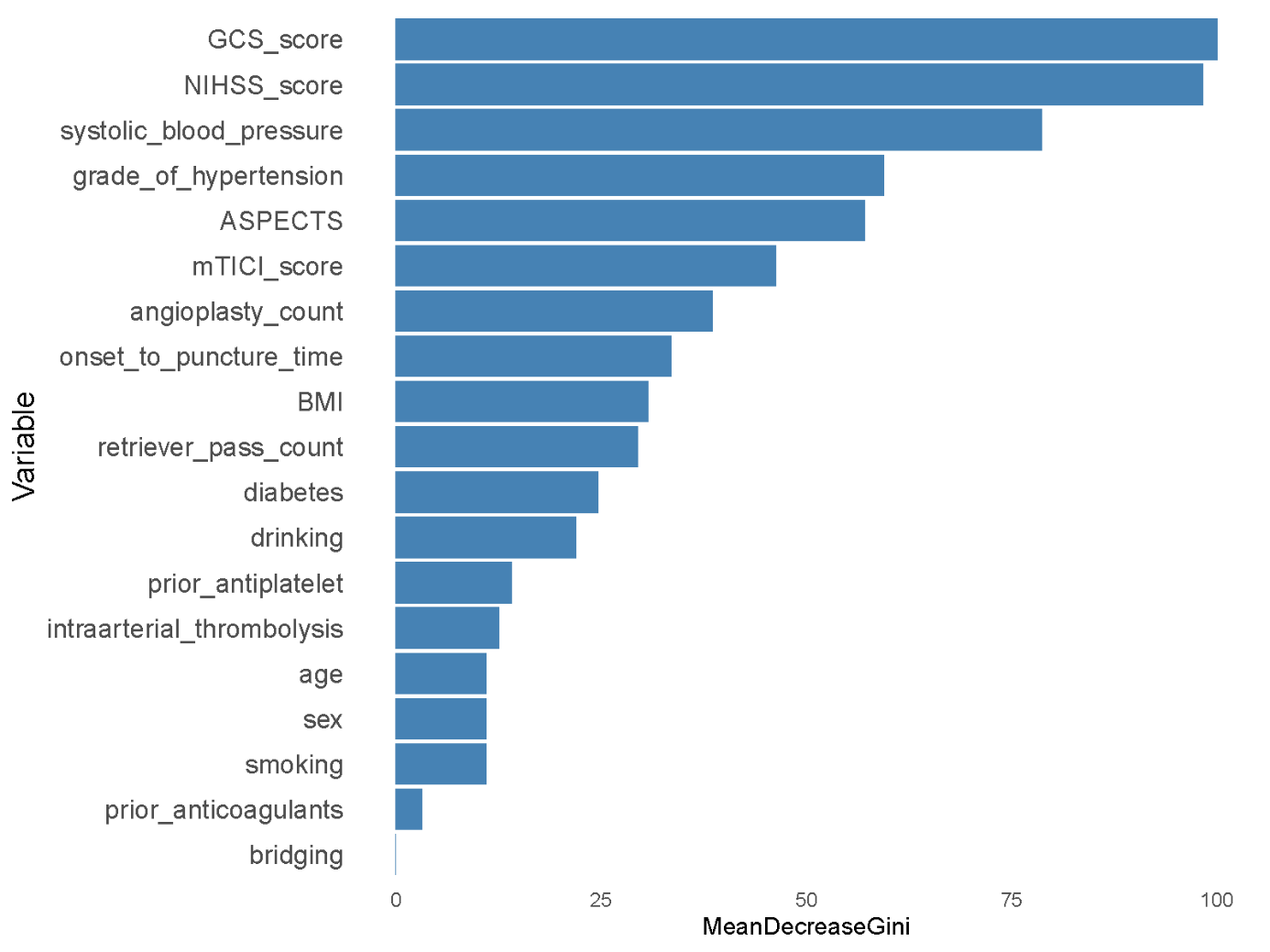
**

**Supplementary Figure 10.** Variable importance plot for the SVM model predicting sICH.

Each bar in the figure represents a feature, with the length of the bar indicating the importance value of that feature. SVM, Support Vector Machine; sICH, symptomatic intracranial hemorrhage; mTICI, modified Thrombolysis in Cerebral Infarction; BMI, body mass index; GCS, Glasgow Coma Scale; NIHSS, National Institutes of Health Stroke Scale; ASPECTS, Alberta Stroke Program Early CT Score.

**Supplementary Table 1.** Summary of Model Performance Metrics.

| **Model** | **AUC(95%CI)** | **adjusted AUC** | **Hosmer-Lemeshow test (*p*-value)** | **sensitivity** | **specificity** |
| --- | --- | --- | --- | --- | --- |
| **LR for ICH** | 0.712(0.641-0.784) | 0.654 | 0.633 | 0.611 | 0.797 |
| **LR for sICH** | 0.830(0.741-0.919) | 0.773 | 0.638 | 0.303 | 0.970 |
| **SVM for ICH** | 0.861(0.812-0.909) | 0.974 | -- | 0.892 | 0.846 |
| **SVM for sICH** | 0.688(0.589-0.786) | 1.000 | -- | 1.000 | 0.921 |

AUC, Area Under the Curve; CI, confidence interval; LR, logistic regression; ICH, intracranial hemorrhage; sICH, symptomatic intracranial hemorrhage; SVM, Support Vector Machine.
