# Supplementary material for: Intracranial hemorrhage prediction in acute ischemic stroke patients with anterior circulation tandem lesions following endovascular thrombectomy
Source: Front Neurol. 2025 Aug 29;16:1598203. doi: 10.3389/fneur.2025.1598203 (PMC12426950; doi:10.3389/fneur.2025.1598203)
Supplement: Supplementary file 2 [file Table_1.docx]

STROBE Statement—checklist of items that should be included in reports of observational studies

|  | Item No. | Recommendation | Page  No. | Relevant text from manuscript |
| --- | --- | --- | --- | --- |
| **Title and abstract** | 1 | (*a*) Indicate the study’s design with a commonly used term in the title or the abstract | 1 | Intracranial Hemorrhage Prediction in Acute Ischemic Stroke Patients with Anterior Circulation Tandem Lesions Following Endovascular Thrombectomy |
|  |  | (*b*) Provide in the abstract an informative and balanced summary of what was done and what was found | 1 | In AIS patients with anterior circulation TL undergoing EVT, diabetes, drinking history, and lower mTICI score independently predict ICH risk, while lower GCS score, lower mTICI score, and lower ASPECTS independently predict sICH risk. The nomograms provide practical tools for individualized risk assessment, aiding clinical decision-making and perioperative management in this high-risk cohort. |
| Introduction | | | |  |
| Background/rationale | 2 | Explain the scientific background and rationale for the investigation being reported | 2 | Worldwide, Acute ischemic stroke (AIS) ranks as a crucial contributor of mortality and disability, with an increasing burden due to aging populations and rising incidence among younger individuals. Tandem lesions (TL) are a more severe form of cerebrovascular disease, characterized by significant stenosis or occlusion in extracranial vessels, coupled with distal intracranial vessel occlusion. Endovascular thrombectomy (EVT) has been proven to be beneficial for AIS patients with TL. However, compared to patients with isolated stenosis, AIS patients with TL have a more complex pathological mechanism, greater difficulty in achieving vessel recanalization, and a worse prognosis. They also face an increased risk of intracranial hemorrhage (ICH) after EVT, along with higher rates of disability and mortality. |
| Objectives | 3 | State specific objectives, including any prespecified hypotheses | 2 | Currently, no predictive model exists for assessing the risk of ICH and sICH after EVT in TL patients. This study aims to construct a machine learning (ML) model to predict the risk of ICH and sICH following EVT in TL patients. We ultimately utilized a nomogram to intuitively display the relative importance of various independent variables in the model, facilitating the control of risk factors and improving patient prognosis. |
| Methods | | | |  |
| Study design | 4 | Present key elements of study design early in the paper | 2 | This study aligns with the guiding principles of the Declaration of Helsinki. As a retrospective study with all data anonymized, it was exempt from the requirement for patient informed consent based on relevant ethical regulations. Informed consent was obtained from all patients prior to undergoing EVT. |
| Setting | 5 | Describe the setting, locations, and relevant dates, including periods of recruitment, exposure, follow-up, and data collection | 2 | We retrospectively collected data from AIS patients with TL who were admitted to Weifang People's Hospital from various regions across the country between June 2020 and November 2024. |
| Participants | 6 | (*a*) *Cohort study*—Give the eligibility criteria, and the sources and methods of selection of participants. Describe methods of follow-up  *Case-control study*—Give the eligibility criteria, and the sources and methods of case ascertainment and control selection. Give the rationale for the choice of cases and controls  *Cross-sectional study*—Give the eligibility criteria, and the sources and methods of selection of participants | 2 | The inclusion criteria were as follows: (1) Diagnosis of AIS by neurologists based on symptoms and imaging examinations; (2) Confirmation of hemodynamically significant obstruction (occlusion or stenosis ≥50%) in the extracranial segment of the anterior circulation large vessel, along with distal intracranial segment occlusion, via magnetic resonance angiography, computed tomography angiography, or digital subtraction angiography; (3) Patients who underwent EVT. The exclusion criteria were as follows: (1) Preoperative existence of ICH; (2) History of coagulation disorders, platelet abnormalities, or thrombocytopenia; (3) Allergy to contrast agents; (4) Severe cardiac, hepatic, renal, or other organ dysfunction; (5) Patients deemed by researchers to interfere with the data interpretation of this study. |
|  |  | (*b*) *Cohort study*—For matched studies, give matching criteria and number of exposed and unexposed  *Case-control study*—For matched studies, give matching criteria and the number of controls per case |  |  |
| Variables | 7 | Clearly define all outcomes, exposures, predictors, potential confounders, and effect modifiers. Give diagnostic criteria, if applicable | 2 | All data were sourced from objective electronic medical records. Patients diagnosed with AIS who presented within the EVT treatment window (24 hours) were immediately enrolled in our hospital's Stroke Green Channel. Preoperative non-contrast head computed tomography (CT) was used to assess the Alberta Stroke Program Early CT Score (ASPECTS), quantifying the infarct core volume before treatment. The decision to perform EVT was made at the discretion of experienced, standardized-trained interventional neurologists, with specific treatment modalities (including stent retriever thrombectomy, balloon angioplasty, or intraarterial thrombolysis) chosen based on the patient's condition. All patients underwent head CT immediately after the procedure and within 24 hours postoperatively. Persistent hyperdensity on non-contrast CT was used to distinguish ICH from contrast extravasation. Dual energy CT was also employed to identify hemorrhage or contrast extravasation. Non-contrast CT was performed using a SIEMENS CT WKL scanner (Siemens Healthcare) with the following parameters: tube voltage 120 kV, tube current 273 mAs, slice thickness 0.6 mm. Dual energy CT was performed using a SIECT DRIVE YX scanner (Siemens Healthcare) with the following parameters: simultaneous imaging at 80 kV/248 mAs and 140 kV/124 mAs, slice thickness 0.6 mm. Raw spiral projection data were reconstructed into three sets: two corresponding to 80 kV and 140 kV, respectively, and a third representing a mixed-energy image simulating conventional 120 kV. Virtual non-contrast image and iodine overlay map were utilized to differentiate hemorrhage from contrast. Two physicians independently reviewed the imaging findings. In cases of disagreement, a third physician determined the final result.  Collected baseline demographic data included body mass index (BMI), age, and sex. Preoperative clinical data included grade of hypertension, diabetes, smoking history, drinking history, prior anticoagulant use, prior antiplatelet use, intravenous thrombolysis (bridging), time from symptom onset to groin puncture (onset to puncture time), National Institutes of Health Stroke Scale (NIHSS) score, Glasgow Coma Scale (GCS) score, admission systolic blood pressure, and ASPECTS. Procedural details included number of stent retriever device passes (retriever pass count), number of balloon angioplasty to dilate intracranial vessel (angioplasty count), intraarterial thrombolysis, and modified Thrombolysis in Cerebral Infarction (mTICI) score. Among the variable “grade of hypertension”, 8 missing values were imputed using the mode (grade 3) among the population with hypertension, as these patients were only described as having hypertension without a specified grade. Missing BMI values (n=6) were imputed using sex-specific averages (males: 24.681; females: 24.097). |
| Data sources/ measurement | 8* | For each variable of interest, give sources of data and details of methods of assessment (measurement). Describe comparability of assessment methods if there is more than one group | *3* | *Clinical outcomes were defined as any ICH and sICH after EVT. Based on the Heidelberg Bleeding Classification, ICH included hemorrhagic infarction1, hemorrhagic infarction 2, parenchymal hematoma 1, parenchymal hematoma 2, parenchymal hematoma remote from infarcted brain tissue, intraventricular hemorrhage, subarachnoid hemorrhage, and subdural hemorrhage. sICH was defined as ICH accompanied by a relevant neurological deterioration, considered as either an increase in NIHSS score by ≥4 points, an increase by ≥2 points in a NIHSS subcategory, or major medical interventions such as intubation or decompressive craniectomy.* |
| Bias | 9 | Describe any efforts to address potential sources of bias | 2 | All data were sourced from objective electronic medical records. Patients diagnosed with AIS who presented within the EVT treatment window (24 hours) were immediately enrolled in our hospital's Stroke Green Channel. Preoperative non-contrast head computed tomography (CT) was used to assess the Alberta Stroke Program Early CT Score (ASPECTS), quantifying the infarct core volume before treatment. The decision to perform EVT was made at the discretion of experienced, standardized-trained interventional neurologists, with specific treatment modalities (including stent retriever thrombectomy, balloon angioplasty, or intraarterial thrombolysis) chosen based on the patient's condition. All patients underwent head CT immediately after the procedure and within 24 hours postoperatively. Persistent hyperdensity on non-contrast CT was used to distinguish ICH from contrast extravasation. Dual energy CT was also employed to identify hemorrhage or contrast extravasation. Non-contrast CT was performed using a SIEMENS CT WKL scanner (Siemens Healthcare) with the following parameters: tube voltage 120 kV, tube current 273 mAs, slice thickness 0.6 mm. Dual energy CT was performed using a SIECT DRIVE YX scanner (Siemens Healthcare) with the following parameters: simultaneous imaging at 80 kV/248 mAs and 140 kV/124 mAs, slice thickness 0.6 mm. Raw spiral projection data were reconstructed into three sets: two corresponding to 80 kV and 140 kV, respectively, and a third representing a mixed-energy image simulating conventional 120 kV. Virtual non-contrast image and iodine overlay map were utilized to differentiate hemorrhage from contrast. Two physicians independently reviewed the imaging findings. In cases of disagreement, a third physician determined the final result. |
| Study size | 10 | Explain how the study size was arrived at | 2 | We retrospectively collected data from AIS patients with TL who were admitted to Weifang People's Hospital from various regions across the country between June 2020 and November 2024. This study aligns with the guiding principles of the Declaration of Helsinki. As a retrospective study with all data anonymized, it was exempt from the requirement for patient informed consent based on relevant ethical regulations. Informed consent was obtained from all patients prior to undergoing EVT. |

Continued on next page

| Quantitative variables | 11 | Explain how quantitative variables were handled in the analyses. If applicable, describe which groupings were chosen and why | 3 | In the baseline data, quantitative variables were expressed as medians and interquartile ranges (based on the "quantile" function), and categorical variables were described by counts and proportions (based on the "prop.table" function). |
| --- | --- | --- | --- | --- |
| Statistical methods | 12 | (*a*) Describe all statistical methods, including those used to control for confounding | 3 | Least Absolute Shrinkage and Selection Operator (LASSO) regression was employed to select optimal predictive features (based on the "glmnet" function in the "glmnet" package). Subsequently, incorporating the features, construct a ML model based on multivariate logistic regression (LR) (based on the "glm" function in the "rms" package). Overdispersion test and Variance Inflation Factor (VIF) values assessed model fit, with VIF ≥5 indicating multicollinearity. Goodness-of-fit of the LR model was further evaluated using the Hosmer-Lemeshow test (*p*-value >0.05 indicates adequate model fit). Additionally, a ML model utilizing Support Vector Machine (SVM) was employed to screen for risk factors influencing the outcome (based on the "svm" function in the "e1071" package). The nomogram of the LR model converted multivariate predictors into a visual scoring system (based on the "nomogram" function in the "rms" package). The discriminative performance was evaluated using Receiver Operating Characteristic (ROC) curves and the Area Under the Curve (AUC), and the relatively adjusted AUC calculated through bootstrap validation (1000 bootstrap resamples) was reported. Calibration of the nomogram was evaluated by calibration curves (1000 bootstrap resamples). |
|  |  | (*b*) Describe any methods used to examine subgroups and interactions |  |  |
|  |  | (*c*) Explain how missing data were addressed | 3 | Among the variable “grade of hypertension”, 8 missing values were imputed using the mode (grade 3) among the population with hypertension, as these patients were only described as having hypertension without a specified grade. Missing BMI values (n=6) were imputed using sex-specific averages (males: 24.681; females: 24.097). |
|  |  | (*d*) *Cohort study*—If applicable, explain how loss to follow-up was addressed  *Case-control study*—If applicable, explain how matching of cases and controls was addressed  *Cross-sectional study*—If applicable, describe analytical methods taking account of sampling strategy |  |  |
|  |  | (*e*) Describe any sensitivity analyses |  |  |
| Results | | | | |
| Participants | 13* | (a) Report numbers of individuals at each stage of study—eg numbers potentially eligible, examined for eligibility, confirmed eligible, included in the study, completing follow-up, and analysed | 4 | Based on the inclusion and exclusion criteria, a total of 200 patients with TL involving anterior circulation large vessel occlusion were enrolled in this study between June 2020 and November 2024 (Figure 1). The cohort comprised 141 males (70.5%) and 59 females (29.5%). After EVT, ICH occurred in 92 patients (46%), and sICH occurred in 24 patients (12%). |
|  |  | (b) Give reasons for non-participation at each stage |  |  |
|  |  | (c) Consider use of a flow diagram |  |  |
| Descriptive data | 14* | (a) Give characteristics of study participants (eg demographic, clinical, social) and information on exposures and potential confounders | 4 | Patient characteristics, including baseline demographics, preoperative clinical data, and procedural details, are presented in Table 1. Correlations between the variables are illustrated in Figure 2. |
|  |  | (b) Indicate number of participants with missing data for each variable of interest |  |  |
|  |  | (c) *Cohort study*—Summarise follow-up time (eg, average and total amount) |  |  |
| Outcome data | 15* | *Cohort study*—Report numbers of outcome events or summary measures over time | *4* | The cohort comprised 141 males (70.5%) and 59 females (29.5%). After EVT, ICH occurred in 92 patients (46%), and sICH occurred in 24 patients (12%). |
|  |  | *Case-control study—*Report numbers in each exposure category, or summary measures of exposure |  |  |
|  |  | *Cross-sectional study—*Report numbers of outcome events or summary measures |  |  |
| Main results | 16 | (*a*) Give unadjusted estimates and, if applicable, confounder-adjusted estimates and their precision (eg, 95% confidence interval). Make clear which confounders were adjusted for and why they were included | 4 | LASSO regression was applied to select features influencing ICH after EVT in TL patients, reducing 19 initial features to 10 potential predictors (Supplementary Figure 1 and 2). The results of the multivariate LR analysis for these predictors are presented in Table 2. Among them, diabetes, drinking history, and low mTICI score were significantly associated with an increased risk of ICH. Based on the LR model, a nomogram for ICH risk prediction was constructed (Figure 3). The final model demonstrated good fit (overdispersion test: p=0.287) and low variable collinearity (VIF range: 1.077-1.734). The Hosmer-Lemeshow test for the model produced a p-value of 0.633. The model exhibited good risk assessment performance (AUC=0.712, 95% CI: 0.641-0.784), with the ROC curve shown in Figure 4. The adjusted AUC via the bootstrap method was 0.654. Calibration curve and decision curve analysis for the ICH nomogram are presented in Supplementary Figure 3 and 4.  LASSO regression identified 8 potential predictors influencing sICH after EVT in TL patients (Supplementary Figure 5 and 6). The results of the LR analysis for these predictors are presented in Table 3. Among them, low GCS score, low mTICI score, and low ASPECTS were significantly associated with an increased risk of sICH. Based on the LR model, a nomogram for sICH risk prediction was constructed (Figure 5), showing good model fit (overdispersion test: p=0.435) and low collinearity (VIF range: 1.022-1.725). The Hosmer-Lemeshow test for the model produced a p-value of 0.638. The model exhibited good risk assessment performance (AUC=0.830, 95% CI: 0.741-0.919), with the ROC curve shown in Figure 6. The adjusted AUC was 0.773. Calibration curve and decision curve analysis for the sICH nomogram are presented in Supplementary Figure 7 and 8. |
|  |  | (*b*) Report category boundaries when continuous variables were categorized |  |  |
|  |  | (*c*) If relevant, consider translating estimates of relative risk into absolute risk for a meaningful time period |  |  |

Continued on next page

| Other analyses | 17 | Report other analyses done—eg analyses of subgroups and interactions, and sensitivity analyses | 4 | SVM models were constructed using all feature variables. The SVM model predicting ICH risk after EVT in TL patients achieved an AUC of 0.861 (95% CI: 0.812-0.909), and its variable importance plot is shown in Supplementary Figure 9. The SVM model predicting sICH risk achieved an AUC of 0.688 (95% CI: 0.589 - 0.786), with the variable importance plot presented in Supplementary Figure 10. A summary table of the performance metrics for each model was provided in Supplementary Table 1. |
| --- | --- | --- | --- | --- |
| Discussion | | | | |
| Key results | 18 | Summarise key results with reference to study objectives | 5 | In the LR model predicting hemorrhage risk after EVT in TL patients, diabetes, drinking history, and low mTICI score were significantly associated with an increased risk of ICH; low GCS score, low mTICI score, and low ASPECTS were significantly associated with an increased risk of sICH. The nomogram of the LR model converted multivariate predictors into a visual scoring system, quantifying variable contributions and linking total scores to outcome probabilities. This tool democratizes predictive analytics, supporting real-time, evidence-based decisions during consultations. |
| Limitations | 19 | Discuss limitations of the study, taking into account sources of potential bias or imprecision. Discuss both direction and magnitude of any potential bias | 6 | We acknowledge several limitations in this study. Firstly, the study was retrospective. Despite the implementation of strict inclusion and exclusion criteria, it remained challenging to entirely eliminate biases among the outcomes. Due to the retrospective nature of the data, imaging data such as precise infarct core volume and relative cerebral blood flow ratios were unavailable; we utilized ASPECTS as a surrogate marker for infarct volume. Additionally, we did not perform dynamic monitoring of perioperative blood pressure and blood glucose levels in TL patients. Secondly, the model was developed using data derived from Chinese patients. Its applicability to populations in other countries has not been validated, necessitating enhanced collaboration with other international stroke centers to improve the model's generalizability. Thirdly, although the creation of predictive models demands 'big data', there is currently no standardized criteria to determine an appropriate sample size. Increasing the sample size or applying data balancing techniques in the future may enhance the model’s predictive performance. |
| Interpretation | 20 | Give a cautious overall interpretation of results considering objectives, limitations, multiplicity of analyses, results from similar studies, and other relevant evidence | 7 | This study demonstrates that in AIS patients with anterior circulation TL, diabetes, drinking history, and low mTICI score significantly increase the risk of ICH following EVT. Meanwhile, low GCS score, low mTICI score, and low ASPECTS significantly increase the risk of sICH. Furthermore, the nomograms constructed using ML models in this study quantify the contribution of variables and link total scores to outcome probabilities, thereby assisting clinicians in rapidly assessing hemorrhage risk for personalized prognosis evaluation and treatment guidance. |
| Generalisability | 21 | Discuss the generalisability (external validity) of the study results |  |  |
| Other information | |  | | |
| Funding | 22 | Give the source of funding and the role of the funders for the present study and, if applicable, for the original study on which the present article is based | 7 | This research was supported by the Graduate Student Research Grant from Shandong Second Medical University, grant number 2024YJSCX016. |

*Give information separately for cases and controls in case-control studies and, if applicable, for exposed and unexposed groups in cohort and cross-sectional studies.

**Note:** An Explanation and Elaboration article discusses each checklist item and gives methodological background and published examples of transparent reporting. The STROBE checklist is best used in conjunction with this article (freely available on the Web sites of PLoS Medicine at http://www.plosmedicine.org/, Annals of Internal Medicine at http://www.annals.org/, and Epidemiology at http://www.epidem.com/). Information on the STROBE Initiative is available at www.strobe-statement.org.
